# Conventional and Emerging Protein Ingredients in Farinaceous Products: Amino Acid Quality, Digestibility, Biological Value, and Functional Performance

**DOI:** 10.1111/1541-4337.70687

**Published:** 2026-10-27

**Authors:** P. Álvarez‐Arteaga, M. A. Lazo‐Vélez, L. Mateo Cordero‐Clavijo, S. Paterson

**Affiliations:** ^1^ NutriOmics Research Group Universidad del Azuay Cuenca Ecuador; ^2^ Department of Bioactivity and Food Analysis Institute of Food Science Research (CIAL, CSIC‐UAM) Madrid Spain; ^3^ Department of Nutrition and Food Science, Faculty of Pharmacy Complutense University of Madrid (UCM) Madrid Spain

**Keywords:** essential amino acids, farinaceous products, protein digestibility, protein enrichment, protein quality, technofunctional properties

## Abstract

The increasing demand for protein‐enriched foods has renewed interest in farinaceous products as widely consumed and technologically adaptable matrices for nutritional improvement. However, protein enrichment cannot be evaluated solely by increases in crude protein content, as nutritional value depends on essential amino acid (EAA) balance, digestibility, biological quality, technofunctional performance, bioactive potential, and interactions within the food matrix. This review critically examines the incorporation of conventional and emerging protein ingredients from animal, plant, insect, microalgal, and macroalgal sources into farinaceous systems, including flours, powders, concentrates, isolates, and hydrolysates. This review synthesizes current evidence through comparative analyses of protein content, EAA profiles, digestibility, protein digestibility‐corrected amino acid score (PDCAAS), digestible indispensable amino acid score (DIAAS), technological functionality, and bioactive properties. Particular attention is given to formulation and processing strategies that improve protein quality while preserving dough or batter performance and consumer‐relevant attributes. In general, the literature indicates that effective protein enrichment requires an integrated approach that combines amino acid complementation, digestibility enhancement, reduction of antinutritional constraints, technological compatibility, and sensory feasibility. This review highlights the need to move beyond protein quantity as the primary benchmark and toward multidimensional quality frameworks for designing nutritionally improved, functional, and acceptable farinaceous foods.

## 1 Introduction

Farinaceous products are widely consumed food matrices and important sources of energy and protein in diets worldwide. Traditionally made from cereal flours and, increasingly, from other flour‐ and starch‐rich ingredients, they are deeply ingrained in the dietary habits and cultural identities of human populations and remain one of the most important sources of readily available energy worldwide (Acevedo‐Pacheco and Serna‐Saldívar 2016).

Due to their affordability, versatility, and popularity, these products provide an attractive platform for incorporating nutritionally important ingredients such as proteins and for delivering bioactive compounds, including phytochemicals, minerals (e.g., Se and Zn), and other agro‐industrial byproducts (Hernández‐López, Abadias, et al. 2023; Cortéz‐Guerrero et al. 2024; Al‐Juhaimi et al. 2024). Moreover, plant‐derived ingredients are often incorporated as complex matrices that provide not only protein but also bioactive compounds, particularly phytochemicals. For example, incorporating fermented green olive pulp has been shown to enhance the phytochemical content and antioxidant capacity of cereal‐based foods (Al‐Juhaimi et al. 2024).

Improving protein nutrition in farinaceous matrices requires more than increasing crude protein levels; protein fortification must translate into improved essential amino acid (EAA) adequacy, digestibility, biological value, technofunctional performance, and sensory quality. This is particularly relevant because cereals, the traditional basis of most farinaceous products, are commonly deficient in specific EAAs, especially lysine, resulting in an incomplete amino acid profile. In addition, cereal‐based matrices may show reduced protein digestibility because of processing conditions and antinutritional factors (ANFs), such as phytates, saponins, and trypsin inhibitors (Joye 2019).

Indeed, these matrices have been extensively studied as vehicles for protein supplementation (Serna‐Saldivar and Lazo‐Vélez 2015; Guardado‐Félix et al. 2019; Boukid et al. 2019; Wang et al. 2023; Cordero‐Clavijo, Martínez, et al. 2025; Cordero‐Clavijo, Espinosa‐Ramírez, et al. 2025). The enrichment of farinaceous products, including bread, cookies, pasta, dumplings, and biscuits, with high‐quality and functional protein sources has gained increasing relevance in response to evolving nutritional demands, global food security challenges, and public health concerns, particularly those related to infants and aging populations (Joint WHO/FAO/UNU Expert Consultation 2007). Therefore, protein enrichment of farinaceous products aligns with both global nutritional needs and current market demand for healthier, more functional, and more sustainable foods. This strategy is also relevant in sports nutrition, where protein quality, amino acid composition, digestibility, and postprandial amino acid availability are key determinants of nutritional functionality (Fanelli et al. 2024).

For this reason, researchers are exploring several protein sources; however, each source has inherent physicochemical characteristics related to its EAA profile, digestibility, biological value, and technofunctionality, which collectively determine the nutritional profile and technological performance of the fortified product (Skendi et al. 2021).

Animal‐derived proteins generally provide a complete EAA profile and high digestibility; however, cost, technological feasibility, allergenicity concerns, dietary restrictions, and sustainability may limit their use (Camargo et al. 2018). In contrast, plant‐based proteins from legumes, pseudocereals, and oilseeds are often more sustainable and may be sensorially accepted, but they are frequently limited in specific EAAs, such as lysine or methionine, and may contain ANFs that reduce nutrient bioavailability. These limitations can be addressed through strategic protein and processing combinations that improve EAA balance (Cordero‐Clavijo et al. 2021), along with processing techniques that reduce ANFs and enhance protein accessibility (Avilés‐Gaxiola et al. 2018). Beyond these conventional sources, emerging protein alternatives, such as insects, microalgae, and macroalgae, are gaining attention as promising options because of their high nutritional quality, functionality, and potential for large‐scale sustainable production (Hernández‐López, Abadias, et al. 2023; Espinosa‐Ramírez et al. 2023; Bottle et al. 2024; Koukoumaki et al. 2024). Nevertheless, their incorporation into farinaceous matrices still requires deeper characterization, particularly regarding digestibility, protein–starch fiber interactions, sensory acceptance, regulatory feasibility, and process optimization.

A comparative analysis of animal, plant‐based, traditional, and emerging protein sources is therefore essential to identify the most suitable ingredients for enriching farinaceous products with nutritional adequacy, acceptable sensory attributes, and functional value. Importantly, many studies on protein‐enriched farinaceous products still emphasize crude protein content as the main indicator of improvement, while less consistently reporting limiting amino acids, digestibility, biological value, or standardized protein quality indices such as the protein digestibility‐corrected amino acid score (PDCAAS) and the digestible indispensable amino acid score (DIAAS). This limits the ability to compare protein sources and to determine whether fortification provides a true nutritional advantage. Accordingly, this review compiles and critically compares the reported effects of diverse protein sources—animal, plant‐based, insect, microalgal, macroalgal, and other emerging alternatives—on EAA profiles, digestibility, biological value, technofunctional properties, and associated functional attributes in farinaceous food matrices. This integrative approach provides a framework (Figure 1) for optimizing both nutritional quality and technological feasibility, addressing the growing demand for functional, sustainable, and tailored foods driven by healthy aging, sports nutrition, and plant‐based dietary trends, while aligning with food industry priorities for sustainability and innovation.

**FIGURE 1:**
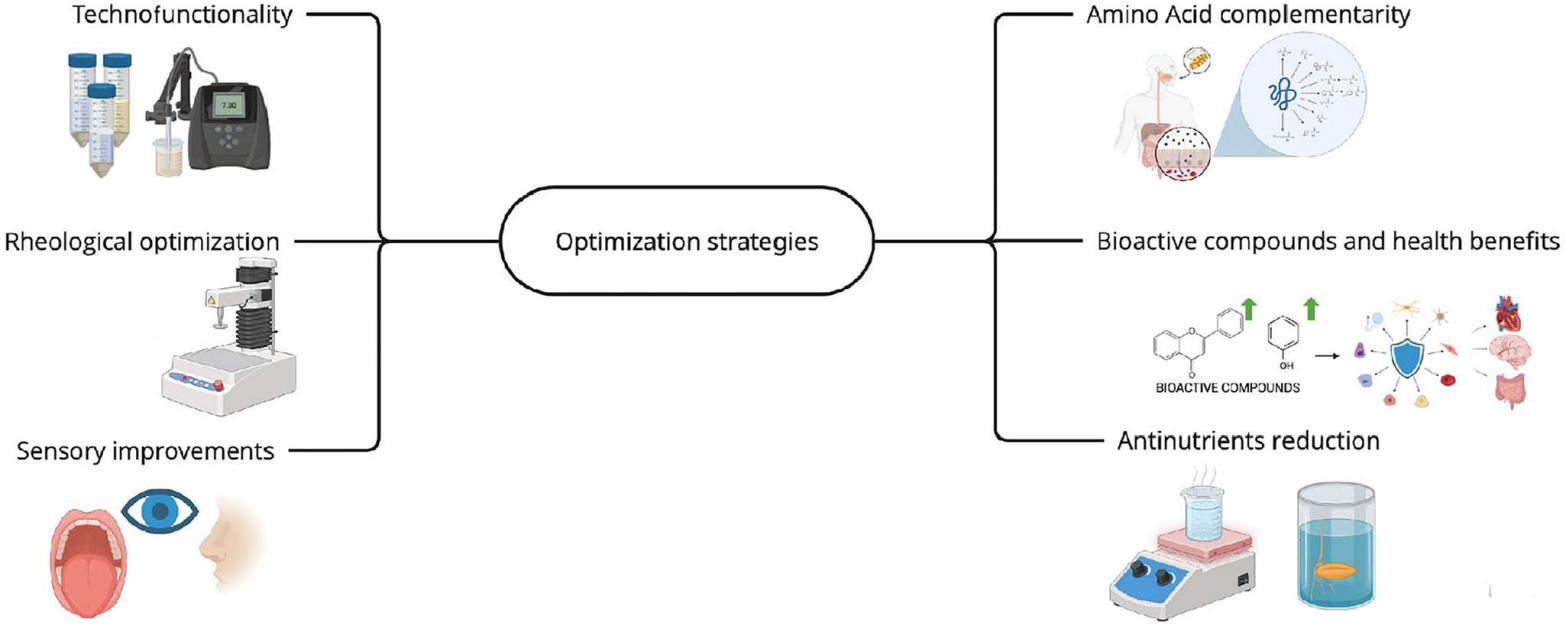
Conceptual framework for the optimization of protein‐enriched farinaceous products across nutritional, technological, sensory, and functional dimensions.

## 2 General Nutritional and Technological Considerations of Protein Incorporation Into Farinaceous Products

To understand how protein sources are incorporated into farinaceous products, it is first necessary to analyze the main nutritional and technological aspects of this process. This should be followed by an examination of the specific characteristics and effects associated with the various protein sources used.

### 2.1 Nutritional Assessments

Although the protein content of flour‐based products is usually evaluated primarily in terms of crude protein—for instance, conventional wheat bread is considered a key reference point with protein levels of around 10%–14%—this parameter alone does not accurately reflect the nutritional value of the finished product (Wang et al. 2023). Therefore, when evaluating protein incorporation, both quantitative and qualitative nutritional criteria, associated with the added protein source, should be considered (G. Wu 2014).

As mentioned above, protein quality must also be considered, particularly the availability and digestibility of EAAs. The EAA profile of wheat bread is deficient in lysine and methionine + cysteine, falling below FAO benchmarks. Because EAA requirements vary with age and physiological demand, the interpretation of protein quality should be linked to target population needs. Although EAA requirements generally decrease with age, the highest relative requirements occur during early life, when EAAs are essential for growth, tissue development, and neurocognitive maturation (Parikh et al. 2022). For this reason, Table 1 presents FAO‐recommended EAA reference values for children aged 1–2 years and adults over 18 years. These values were used to compare the EAA profiles of the analyzed matrices. Conventional wheat bread was also included as a reference matrix to contextualize the nutritional improvement achieved through protein fortification.

**TABLE 1:** Comparative protein of enriched farinaceous products: Essential amino acid adequacy, digestibility, biological value, and PDCAAS/DIAAS.

Matrix	Protein source	Essential amino acids (EAAs) (g AA/100 g protein)	Leucine	Valine	Isoleucine	Lysine	Methionine + cysteine (SAA)	Phenylalanine + tyrosine (AAA)	Threonine	Tryptophan	Histidine	Protein digestibility	PDCAAS or DIAAS	Reference
Reference: FAO data (1–2 years)			6.3	4.2	3.1	5.2	2.5	4.6	2.7	0.7	1.8			Joint WHO/FAO/UNU Expert Consultation 2007
Reference: FAO data (>18 years)			5.9	3.9	3.0	4.5	2.2	3.8	2.3	0.6	1.5			
Control: Wheat bread			6.8	4.4	4.2	2.6	1.00	8.2	2.8	1.2	2.3			Delmi Bouras et al. 2022
Animal	Wheat bread	Whey protein: Pure powder	↑17.24	↑9.47	↑10.29	↑16.15	↑3.74	↓5.73	↑12.00	↑2.88	↑3.19	—	—	Delmi Bouras et al. 2022
	Banana cake	Whey protein: Isolated and hydrolyzed (92% protein)	↓0.184^a^	↓0.11^a^	↓0.092^a^	↓0.108^a^	ND	↓0.99 (PHE)^a^	↓0.139^a^	ND	↓0.039^a^	—	—	Camargo et al. 2018
	Wheat bread	Hydrolyzed fish‐whey blend	6.12^a^	4.89	↓3.06^a^	↑3.60^a^	↑3.59	7.44	3.27	1.03	ND	—	—	Stănciuc et al. 2018
	Cheese cake	Milk protein: Concentrate	↓5.50^a^	4.0^a^	↓2.80^a^	↑4.0^a^	↑2.50	↓4.70	2.7	↓0.70	ND	—	DIAAS: 84	Deinychenko et al. 2020
	Wheat bread	Salmon: Powder	6.5	3.75^a^	3.46	2.69^a^	↑4.38	↓6.38	3.05	ND	1.94	—	—	Desai et al. 2018
Plant	Wheat bread	Yellow lupin polo: Flour	7.4	5.2	4.1	↑3.70^a^	↑3.30	6.9	2.8	↓0.80	ND	50.5	PDCAAS: 62.5	Kłobukowski et al. 2021
	Egg‐free cake	Quinoa protein: Isolate	5.76^a^	4.48	↑9.64	↑11.17	↑4.75	↓4.42^a^	ND	↑5.99	↑4.78	—	—	Mohamed et al. 2025
	Gluten‐free cake	Quinoa flour and encapsulated spirulina powder (QES)	↓5.45^a^	4.14^a^	3.07^a^	↑4.14^a^	↑2.68	6.01	↑3.67	ND	↓1.61^a^	—	—	Peñalver and Nieto 2024
		Amaranth flour and encapsulated spirulina powder (AES)	5.67^a^	4.01^a^	3.27	↑4.52^a^	↑2.51	↓5.18	↑3.69	ND	1.95			
		Brown rice flour and spirulina powder (BRES)	7.46	4.83	4.47	↑5.81	↑2.67	7.75	↑5.04	ND	2.65			
	Wheat bread	Brown rice flour and spirulina powder (BRES)	6.5	4.46	4.17	2.72^a^	↑3.39	8.34	2.81	1.16	1.94	86	PDCAAS: 65	Peñalver and Nieto 2024
	Wheat bread	Faba bean: Flour Carob bean: Flour Gluten: Flour	7.03	4.38	3.77	↑3.90^a^	↑2.58	6.69	3.12	↓0.78	2.23	—	PDCAAS: 77	Hoehnel et al. 2020
Alternative	Wheat bread	*Tenebrio molitor* full fat (FFTM): Flour	7.11	4.83	3.98	↑3.45^a^	↑1.45^a^	↓4.80	3.27	1.13	↑2.46	85.9	PDCAAS: 61.72	Bottle et al. 2024
	Wheat bread	Cricket: Sourdough/powder	8.5	ND	4.53	↑3.42^a^	ND	↓4.73 + TYR	↑7.79	ND	1.70^a^	—	—	Osimani et al. 2018
	Wheat bread	Mealworm: Sourdough/defatted powder	↑10.50	↓1.60^a^	5.2	2.70^a^	↑8.30 + CYS	↓6.60	↑9.40	1.3	2.3	—	—	Roncolini et al. 2019
	Crackers	1.5% *Spirulina*	16.5	11.6	11.6	0.1	—	—	ND	ND	—	—		Hernández‐López, Abadias, et al. 2023
	Grissini	3.5% *C. vulgaris*	5.1	6.8	4	0.2	—	—	0.2	0.2	—	—		
	Wheat bread	*N. gaditana*	8.6	5.3	4.1	6.3	3	7.9	4.2	1.9	1.9		DIAAS: 73	
	Wheat bread	Ethanol‐treated *N. gaditana*	9.9	5.3	4.9	5.3	3.3	9.4	4.3	2.3	2.3		DIAAS: 84	
	Wheat Bread	*T. chuii*	8.5	5.9	4	6.2	4.4	8.8	4.6	1.8	1.7		DIAAS: 54	Nunes et al. 2020
		Ethanol‐treated *T. chuii*	8.9	6.1	4.8	4.9	3.7	9.2	4.8	2.1	1.8		DIAAS: 78	
		*C. vulgaris*	9.4	6.1	4	5.7	3.3	9.4	4.7	1.9	1.8		DIAAS: 69	
		Ethanol‐treated *C. vulgaris*	8	4.5	4.9	5.3	3.3	9.4	4.3	2.3	2.3		DIAAS: 73	
		6% *Spirulina*	0.52	1.37	0.22	0.36	0.87	1.42	0.43	0.39	—	—	—	Montevecchi et al. 2022
		2% *Spirulina*	4.49	3.15	2.37	2.98	1.06 + MET	2.98 + PHE	2.48	—	1.23	—	—	

*Note*: ↑ denotes an increase of ≥20% relative to the control group, and ↓ indicates a decrease of ≥20% relative to the control group.

^a^ Fails to meet the minimum requirement established by the FAO for children aged 1–2 years.

Abbreviation: ND, not determined.

Proteins contain both EAAs and nonessential amino acids. While nonessential amino acids can be synthesized endogenously, EAAs must be obtained through the diet. However, only a fraction of ingested amino acids is effectively digested, absorbed, and used, making protein quality assessment essential when evaluating fortified farinaceous products.

Protein quality is commonly assessed using complementary parameters, including amino acid composition, limiting amino acid, protein digestibility, biological value, PDCAAS, and DIAAS (Wolfe et al. 2016). Biological value reflects the efficiency with which absorbed amino acids are retained and used by the organism, although it does not fully account for matrix effects, processing‐induced interactions, or differences in amino acid digestibility. Protein digestibility, particularly ileal digestibility, provides a more direct estimate of amino acid availability before microbial metabolism in the distal intestine (Boye et al. 2012). PDCAAS and DIAAS are standardized indices used to integrate amino acid composition and digestibility. However, they differ in physiological relevance: PDCAAS is based on total‐tract crude protein digestibility and truncates values at 1.0, whereas DIAAS is based on standardized ileal digestibility of individual indispensable amino acids and is therefore considered more discriminating for high‐quality proteins (Mathai et al. 2017). Consequently, when available, DIAAS provides a stronger basis for comparing the nutritional relevance of protein‐enriched matrices. Returning to the example of bread, its PDCAAS score of 0.44 (Bottle et al. 2024) suggests that its protein quality is relatively poor compared with enriched sources. This low score is mainly due to the lysine deficiency of wheat proteins, which limits the amino acid score (AAS), while gluten digestibility may be further reduced by compact, protease‐resistant structures and residual protease inhibitors.

Protease inhibitors, particularly trypsin inhibitors, are generally heat‐labile and can be partially or extensively inactivated during thermal processing through protein denaturation (Cordero‐Clavijo et al. 2024). Other ANFs, including phytates, tannins, and certain oligosaccharides, may also be reduced through processing strategies such as soaking, germination, fermentation, and enzymatic treatment, thereby improving protein digestibility and nutrient bioavailability (Yılmaz Tuncel et al. 2025).

### 2.2 Technological Assessments

In addition to increasing protein content, protein ingredients modify the technological behavior of farinaceous matrices because of their molecular structure, hydration properties, surface activity, and interactions with starch, gluten, lipids, and fiber. These technofunctional properties largely determine dough handling, gas retention, crumb structure, texture, color development, and overall sensory quality (De Angelis et al. 2024). Therefore, the success of protein enrichment in baked products depends not only on nutritional improvement but also on whether the incorporated protein integrates into the matrix without disrupting its structure or consumer acceptability.

Thermal processing is a critical determinant of EAA preservation in baked systems. Although baking can induce protein denaturation and Maillard reactions, whey proteins may retain considerable nutritional value under certain conditions. Their globular structure partially unfolds at moderate temperatures while preserving peptide bonds, and the gluten–starch matrix can limit heat penetration into the product core. Thus, although surface temperatures may reach high values during baking, internal temperatures generally remain below 100°C, which helps preserve amino acid integrity. In addition, Maillard reactions in low‐moisture systems may preferentially involve available reducing sugars rather than whey lysine, with studies reporting EAA retention above 90% under specific baking conditions (Stănciuc et al. 2018). Nevertheless, this protective effect should not be generalized across all products, since formulation, moisture, pH, sugar content, baking time, and protein format can strongly influence lysine availability and digestibility.

Specific volume is one of the most relevant indicators of baking performance because it reflects gas retention and the dough or batter's ability to maintain a stable structure during proofing and baking. For example, cake prepared with quinoa protein isolate showed favorable performance, as its protein fractions may generate interaction sites with gluten and gelatinizing starch through hydrogen bonding and hydrophobic interactions, increasing viscoelasticity and stabilizing air cells during baking (Mohamed et al. 2025). Therefore, moderate incorporation levels can improve structural performance when the added proteins contribute to foam stabilization or matrix reinforcement rather than competing excessively with gluten.

Texture is another critical quality parameter, particularly in protein‐enriched products where excessive protein or fiber incorporation often increases hardness. Bread enriched with milk protein concentrate (MPC) showed improved softness, likely because caseins have a high water‐binding capacity (WBC) and can form soft gels that co‐structure with gluten through electrostatic interactions and calcium‐mediated salt bridges (Shabunina et al. 2023). This behavior can reduce excessive water competition and help preserve crumb tenderness. In contrast, plant proteins and fiber‐rich ingredients may increase firmness when incorporated at high levels, because they compete for water, dilute gluten, and interfere with gluten network development. As Dhen et al. (2018) reported, excessive fiber and plant protein can impair gluten development and lead to volume collapse. Therefore, ingredient functionality must be evaluated together with substitution level, since the same protein source may be beneficial at moderate levels but detrimental when incorporated beyond the matrix tolerance.

Protein enrichment strongly affects color development, especially when ingredients contain high levels of amino groups, reducing compounds, pigments, or chitin. Formulations containing more than 12% apricot kernel flour or 10% mealworm powder displayed darker hues and more reddish‐yellow tones, associated with accelerated nonenzymatic browning reactions and pigment contribution, leading to lower L* values and higher a* and b* values (Dhen et al. 2018; Roncolini et al. 2019). Although darker color may be acceptable or even desirable in some baked products, it can also reduce consumer acceptance when it suggests overbaking, oxidation, or unfamiliar ingredients. This is particularly relevant for insect‐ and algae‐enriched products, where natural pigments and lipid oxidation products may alter both appearance and flavor.

Water absorption and hydration behavior are central to the functionality of protein‐enriched doughs. Legumes and pseudocereals generally exhibit high water retention, solubility, emulsifying, foaming, and gelling capacities, which may support dough structure and product softness when properly balanced. However, excessive water absorption can reduce free water availability for gluten development and starch gelatinization, thereby compromising gas retention and final volume. This explains why protein‐rich ingredients often require formulation adjustments, including changes in water addition, mixing time, hydrocolloid use, or pretreatment of the protein ingredient. At the starch level, protein incorporation can directly modify both gelatinization and retrogradation through competition for water and intermolecular interactions. Proteins with high WBC may reduce the amount of water available for starch granule swelling during heating, altering gelatinization behavior. In parallel, denatured proteins can interact with amylose and amylopectin through hydrogen bonding and hydrophobic interactions, modifying the continuity and rigidity of the starch–protein network (Hu et al. 2026). During cooling and storage, protein structure and degree of hydrolysis become particularly relevant, since smaller peptides may interfere with starch‐chain reassociation and crystallization, reducing retrogradation, as observed for soy protein hydrolysates incorporated into wheat‐based cookies (Knežević‐Jugović et al. 2023). Thus, the effect of protein enrichment on starch functionality depends on protein concentration, conformation, degree of hydrolysis, and affinity for water and starch.

Sensory acceptance remains the ultimate criterion for technological feasibility. Among animal protein formulations, MPC‐enriched bread received the highest preference, whereas among plant‐based products, cake containing 8.6% quinoa protein isolate was the most highly rated by consumers (Mohamed et al. 2025; Shabunina et al. 2023). These examples show that successful protein enrichment is usually achieved when nutritional improvement is accompanied by acceptable texture, volume, color, flavor, and mouthfeel. Across the reviewed studies, the most feasible substitution range was generally between 5% and 20%, although this interval should not be considered universal. The optimal level depends on the protein source, ingredient format, target product, processing conditions, and desired nutritional claim (Figure 3).

## 3 Conventional and Emerging Protein Ingredients

A wide variety of protein sources, including those derived from animals (Table 2), plants (Table 3), and nontraditional or alternative sources (Table 4), have been incorporated into farinaceous food matrices. The selection of these sources depends on whether the aim is to increase protein content, improve technological quality, or meet sensory requirements. However, improvements to these characteristics depend not only on the amount of protein incorporated but also on the format of the ingredient (e.g., unprocessed or defatted flours, protein concentrates, isolates, or hydrolysates) (Lazo‐Vélez et al. 2015), its EAA profile, digestibility, PDCAAS or DIAAS, bioactive potential, and the technological interactions occurring within the food matrix (Hoehnel et al. 2020). The following sections therefore compare the nutritional, technological, and bioactive effects of different protein sources when incorporated into flour‐based food matrices.

**TABLE 2:** Animal‐derived proteins in farinaceous products: Ingredient form, substitution level, protein enrichment, and technological performance.

Matrix	Protein source	Substitution rate (%)	Protein content (%)	Relative protein increase (%)	Observations	Reference
Wheat bread	Whey protein: Pure powder	2.50–20	12.09	20.9	Decrease in the baking and rheological characteristics.	Delmi Bouras et al. 2022
			12.96	29.6		
			20.45	104.5		
			32.85	228.5		
Gluten‐free cookies	Whey protein: Powder (88.96% protein)	15–20	9.19	171	Pea protein was the type that presented the fewest differences compared to the control cookie.	Deinychenko et al. 2020
			14.97	341.6		
Banana cake	Whey protein: Isolated and hydrolyzed (92% protein)	10–30	12.15	44.47	Whey protein can be added to cakes with no loss to physical, chemical, or sensory qualities, aiming at technological and nutritional improvements.	Camargo et al. 2018
			18.06	114.7		
			20.93	148.87		
Wheat bread	Milk protein: Concentrate	9	11.6	54.66	Positive effect on the volume yield of bread.	Shabunina et al. 2023
Cheese cake	Milk protein: Concentrate	84	26.98	68.40	Effectiveness of MPC usage as a substitute for cottage cheese in culinary product making.	Deinychenko et al. 2020
Wheat bread	Raw yoghurt	30–50	14.9	76	With curd cheese (Cc) additions of 30 g or more, a significant increase in crumb firmness and aging kinetics was observed.	Graça et al. 2019
			15.8	87.2		
	Curd cheese	30–50	17.1	102.6		
			20.3	140.52		
Gluten‐free cookies	Egg white protein: Powder (84.83% protein)	15–30	9.08 14.34	167.8 323	The choice of the correct protein is essential, as certain types of protein with suboptimal characteristics cause alterations in dough.	Sahagún and Gómez 2018
Biscuit	Hydrolyzed fish–whey blend	20	15.1	67.88	Increasing biological value and maintaining their organoleptic characteristics.	Klyuchko et al. 2022
Wheat bread	Fish residue: Flour	4.2–16.8	10.2	21.4	Sensory acceptance is better than or as good as that of a standard bread formulation without fish flour.	Bastos et al. 2014
			12.1	44		
			13.1	56		
			14.4	69		
Wheat bread	Salmon: Powder	5–15	16.3 18.2 20	17.18 30.84 43.78	SFP bread inhibits starch digestion by increasing the protein matrix around the starch granules, thereby lowering the release of reducing sugars. The antioxidant capacity of the bread was significantly enhanced.	Desai et al. 2018
Wheat bread	Tilapia waste: Powder	2.5–20	8.71	28.46	Intention to consume and to purchase enriched bread can be maintained with levels <12.17% and <6.86%.	Monteiro et al. 2018
			10.83	59.73		
			12.01	77.13		
			14.36	111.79		
			16.28	140.11		

Abbreviations: Cc, curd cheese; MPC, milk protein concentrate; ND, not determined; SFP, salmon fish powder.

**TABLE 3:** Plant‐based proteins in farinaceous products: Ingredient form, substitution level, protein enrichment, and technological performance.

Matrix	Protein source	Substitution rate (%)	Protein content (%)	Relative protein increase (%)	Observations	Reference
Tortillas	Soybean: Defatted flour	6	11.52	25.62	Improved the protein:calorie ratio, lysine and tryptophan contents, PER, nitrogen retention values, and PDCAAS.	Acevedo‐Pacheco and Serna‐Saldívar 2016
Bread			11.55	24.2		
Wheat tortillas			9.84	23.77		
Wheat bread	Soy: Protein concentrate	5–15	8.33	9.46	Breads showed a stronger dough structure, higher volume, and softer crumb.	Belc et al. 2021
			9.28	21.94		
			10.2	34.03		
Mold bread	Wheat: Flour Defatted soy: Flour Whey protein: Concentrate	WF: 88.8 DSF: 8.2 WPC: 3	12.6	31.25	The optimized mixture (88.8% WF, 8.2% DSF, and 3.0% WPC) resulted in a high‐protein product with an increase in dietary fiber content and improved calcium levels, without affecting mold bread acceptability.	Erben and Osella 2017
Wheat bread	Cassava: Flour Soybean: Flour	CF: 10 SF: 19	17.5	ND	With controlled processing of soybean and cassava flours, and by maintaining wheat flour at around 70% and a high proportion of soybean flour (about 17%) in the blend, bread with a high nutrient content and soybean‐derived bioactive compounds can be produced.	Ayele et al. 2017
Wheat‐rye bread	Yellow lupin Juno: Flour	5	10.1	11.97	The greatest increase in effective protein (EP) content, the chemical score (CS), the protein digestibility–corrected amino acid score (PDCAAS), and the essential amino acid index (EAAI) was achieved when the bread was enriched with flour from the Polo cultivar.	Kłobukowski et al. 2021
	Yellow lupin polo: Flour		10.5	16.4		
	Yellow lupin Legat: Flour		10.8	19.73		
	Yellow lupin Markiz: Flour		11	21.95		
Wheat bread	Lupin: Debittered flour	10–20	18.5	ND	Offers similar nutritional enhancement as debittered flour, improves dough water absorption, and yields a softer bread with a more acidic, acceptable flavor.	Prieto‐Vázquez Del Mercado et al. 2022
			20			
			20.4			
	Lupin: Fermented flour	10–20	17.6			
			19.9			
			20.1			
Wheat bread	Lupin: Flour Sacha inchi: Flour	CH: 4 SI: 9	19.83	36.19	Lysine remained the limiting amino acid; the formulation enhanced PDCAAS and maintained favorable textural and sensory properties.	Álvarez‐Quinteros et al. 2025
		CH: 6 SI: 7	20.26	39.14		
		CH: 10 SI: 5	20.95	43.88		
		CH: 20	21.97	50.89		
Egg‐free cakes	Quinoa protein: Isolate	2.8–8.6	6.05	−6.2	A potential egg replacer in bakery products designed for people with an allergy to eggs.	Mohamed et al. 2025
			6.6	2.32		
			6.82	5.73		
			7.9	22.48		
Wheat bread	Amaranth: Flour	5–15	12.61	3.61	Amaranth flour substitution resulted in the highest total phenolic content.	Nasir et al. 2020
			12.96	6.49		
			13.11	7.72		
Wheat bread	Amaranth: *A. spinosus* flour Amaranth: *A. hypochondriacus* flour	25–50	18.1	9.69	Substitution with amaranth could supply a high percentage of the DRI for Zn and Fe in adults, but the high amount of phytates in these formulations could inhibit mineral availability. Consumers preferred the bread with 25% replacement with whole *A. hypochondriacus*.	Miranda‐Ramos et al. 2019
			18.4	11.51		
		25–50	17.6 19	6.66 15.15		
Wheat bread	Apricot kernel: Flour	4–24	12.33	12.91	>12% is not recommended; it results in reduced loaf volume, a brown color, a hard texture, and an undesirable taste. Replacement recommendation: 8%.	Dhen et al. 2018
			12.5	14.46		
			14.24	30.4		
			15.51	42.03		
Wheat bread	Hemp: Sourdough/flour	5–15	13	9.24	Use of the hemp sourdough as an ingredient in breadmaking enhanced the main nutritional features of the baked goods.	Nionelli et al. 2018
			14.1	18.48		
			15.2	27.73		
Wheat bread	Pea: Flour	30	15.6	20.23	At 30% substitution, toasted pea flour produced bread with specific volume and loaf density comparable to the wheat control, while all pea flour treatments increased bread protein content.	Millar et al. 2019
	Pea: Germinated flour		16.1	28.57		
	Pea: Toasted flour		16	23.8		
Wheat bread	Pea: Protein concentrate	5–15	9.34	22.73	Pea protein concentrate produced greater total crumb color change than soy protein concentrate.	Belc et al. 2021
			11.49	50.98		
			13.02	71.09		
Gluten‐free cookies	Pea protein (78.13% protein)	15–30	8.34	113.84	Pea protein was the type that presented the fewest differences compared to the control cookie.	Sahagún and Gómez 2018
			13.3	241.02		
	Potato protein (89.87% protein)	15–30	9.58	145.64		
			15.7	302.56		
Wheat bread	Faba bean: Flour Carob bean: Flour Gluten: Flour	10	24.8	60	Adequate baking, sensory, and antioxidant properties.	Ayele et al. 2017
		5				
		2.5				

Abbreviations: CF, cassava flour; CH, Lupin; CS, chemical score; DRI, dietary reference intake; DSF, defatted soy flour; EAAI, essential amino acid index; ND, not determined; PDCAAS, protein digestibility‐corrected amino acid score; PER, protein efficiency ratio; SF, soybean flour; SI, Sacha inchi; WF, wheat flour; WPC, whey protein concentrate.

**TABLE 4:** Emerging alternative proteins in farinaceous products: Ingredient form, substitution level, protein enrichment, and technological performance.

Matrix	Protein source	Substitution rate (%)	Protein content (%)	Relative protein increase (%)	Observations	Reference
Wheat bread	*A. domesticus* full fat (FFAD): Flour	10	18.7	27.2	DFAD was the best option for enhancing the nutritional value of wheat bread in terms of protein concentration and PDCAAS without detrimentally affecting the functional qualities	Bottle et al. 2024
	*A. domesticus* partially defatted (PDAD): Flour		18.3	24.5		
	*A. Domesticus* completely defatted (DFAD): Flour		19.7	34.01		
	*T. molitor* full fat (FFTM): Flour		17.5	19.04		
	*T. molitor* partially defatted (PDTM): Flour		18.7	27.2		
	*T. molitor* completely defatted (DFTM): Flour		18.8	27.9		
Wheat bread	Cricket: Powder	10–30	26.89	83.04	The high load of spore‐forming bacteria in the bread loaves containing cricket powder, as revealed by the microbiological analyses, highlighted potential safety issues for consumers.	Osimani et al. 2018
			27.72	88.69		
	Cricket: Sourdough/powder	10–30	25.39	72.83		
			26.13	77.87		
Wheat bread	Mealworm: Defatted powder	5–10	10.54	18.29	The proposed breadmaking technology is at TRL 4, with prototypes ready for testing in a lab environment.	Roncolini et al. 2019
			11.55	29.62		
	Mealworm: Sourdough/defatted powder	5–10	9.8	9.98		
			11.62	30.41		
Wheat bread	Grasshopper: Powder	10–20	14.8	32.14	Grasshopper powder can be incorporated into baked products in order to increase their nutritional value without damaging their textural and sensory attributes.	Haber et al. 2019
			17.4	55.35		
	Grasshopper: Defatted powder	20	18.1	61.6		
Wheat bread	Cinereous cockroach: Flour	10	22.7	134.5	Presented satisfactory sanitary conditions and can therefore be used in human food with a proper chemical composition, showing a good nutritional profile due to the amino acid.	De Oliveira et al. 2017
Wheat bread	Quinoa flour and spirulina powder (QNES)	9.50 1.30	5.55	87.5	Nonencapsulated spirulina bread significantly increased its nutritional richness, particularly with regard to proteins, amino acids, folates, essential minerals, and fiber. The encapsulation of spirulina proved successful, significantly increasing antioxidant capacity, phenolic compound content, and sensory acceptance.	Miranda‐Ramos et al. 2019
	Quinoa flour and encapsulated spirulina powder (QES)	9.50 1.30	5.34	80.4		
	Control with quinoa flour (Q)	9.5	5.43	83.44		
	Bread with sourdough: Brown rice flour spirulina powder (BRNES)	9.50 1.30	5.35	80.74		
	Brown rice flour and spirulina powder (BRES)	9.50 1.30	5.28	78.37		
	Control with brown rice flour (BR)	9.50	5.35	80.74		
	Amaranth flour with spirulina powder (BRNES)	9.50 1.30	5.5	85.8		
	Amaranth flour and encapsulated spirulina powder (AES)	9.50 1.30	5.29	78.71		
	Control with amaranth flour (A)	9.5	5.4	82.43		
Pasta	*A. platensis*	2–15	65.71	77.47	The protein content expected based on the proportion of *A. platensis* used in each formulation was compared with the protein content measured after cooking, confirming good nutrient retention with no substantial losses.	Koli et al. 2022
Crackers and grissini	*Spirulina*, *Chlorella*, and *Tetraselmis*	1.5–3.5	ND	No increase	In terms of amino acid content, *Spirulina* crackers were shown to be rich in alanine, aspartate, and tryptophan, while *Chlorella* grissini stood out for being particularly rich in isoleucine, leucine, lysine, and valine.	Hernández‐López, Abadias, et al. 2023
Four wheat flours	*Spirulina*	1.5–2.5	65	12 to 20	Adding 2.5% Spirulina increases bread protein content by approximately 1.5 percentage points, representing a 15% relative protein enhancement across all flour types tested.	Hernández‐López, Alamprese, et al. 2023
Gluten‐free bread	*T. chuii*	1–4	47.7	ND	*T. chuii* 4% recovers dough structure.	Nunes et al. 2020
Gluten‐free bread	*T. chuii*	1–4	59.5	16 (17 in ethanol‐treated *T. chuii*)	Ethanol treatment transformed the technological profile by purifying microalgal proteins while removing lipids and pigments.	M. W. Qazi et al. 2022
	*C. vulgaris*		47.8	16 (25.3 in ethanol‐treated *C. vulgaris*)		

Abbreviations: A, control with amaranth flour; AES, amaranth flour with encapsulated spirulina powder; BRES, brown rice flour with encapsulated spirulina powder; BRNES, brown rice flour with nonencapsulated spirulina powder; DFAD, defatted *Acheta domesticus* flour; DFTM, defatted *Tenebrio molitor* flour; FFAD, full‐fat *Acheta domesticus* flour; FFTM, full‐fat *Tenebrio molitor* flour; ND, not determined; PDAD, partially defatted *Acheta domesticus* flour; PDCAAS, protein digestibility‐corrected amino acid score; PDTM, partially defatted *Tenebrio molitor* flour; Q, control with quinoa flour; QES, quinoa flour with encapsulated spirulina powder; QNFS, quinoa flour with nonencapsulated spirulina powder; R, control with brown rice flour; TRL, technology readiness level.

### 3.1 Animal‐Derived Protein

Animal‐derived protein ingredients, including eggs, milk, and their derivatives (cheese, yogurt, and whey), have been widely used in bakery and pastry products because of their technofunctional properties, which contribute to desirable sensory attributes such as flavor, color, and texture. While their use was initially focused primarily on these technological functions, since the 20th century they have also been investigated as sources of nutritional enrichment, particularly for increasing protein content and promoting the incorporation of bioactive compounds into farinaceous products.

Furthermore, the development of the food industry has significantly expanded the availability of protein ingredients for the enrichment of farinaceous products. These include milk‐ and egg‐derived ingredients with different degrees of processing, such as powder, protein concentrates, isolates, and hydrolysates. In parallel, the interest in diversifying protein sources has stimulated the evaluation of alternative animal matrices, including byproducts and derivatives from aquaculture and fisheries species such as tilapia and salmon. Collectively, these ingredients represent new opportunities to enhance the protein content and functional value of farinaceous products, while also contributing to the valorization of food industry co‐products and the utilization of alternative resources. Table 2 summarizes the main applications and effects reported for these protein sources.

#### 3.1.1 Whey Protein

Whey proteins, mainly β‐lactoglobulin, α‐lactalbumin, and lactoferrin, are widely recognized for their high biological value and rapid digestibility. Whey supplementation has been shown to stimulate muscle protein synthesis more effectively than lower‐quality protein sources (Kanda et al. 2013); therefore, its incorporation into wheat bread could enhance postprandial amino acid availability. Whey protein powder showed the highest compliance with EAA requirements, exceeding FAO reference values by more than 20% and representing the only evaluated animal‐derived source that fulfilled the lysine requirement reported in Table 1. This is nutritionally relevant because lysine is one of the most limiting EAAs in cereal‐based formulations (Diana et al. 2014). However, this potential physiological advantage depends on the preservation of EAA availability during processing and digestion, rather than on the intrinsic quality of whey protein alone.

When whey protein isolate (WPI) or hydrolyzed whey protein was incorporated at a substitution rate of up to 30%, the resulting banana cake contained 21% crude protein, representing a 148% increase compared to the control (Camargo et al. 2018). This increase in protein content was observed alongside adequate rheological properties of the dough. However, despite the high intrinsic quality of whey protein, the EAA concentrations of the enriched cakes remained below the recommended minimum levels. These effects were found to be related to the degree of hydrolysis of the whey proteins. Because a commercially hydrolyzed isolate was used (Table 2), a limited degree of hydrolysis may have been insufficient to release all the amino acids in bioavailable forms. Incomplete hydrolysis can produce high‐molecular‐weight peptides that can form rigid structural networks, thereby limiting the release of free amino acids during digestion. Conversely, depolymerization resulting from hydrolysis reduces the molecular weight of proteins, which can enhance moisture retention, foaming, and foam stability within the matrix. This helps to prevent dry or chewy textures (Camargo et al. 2018).

Similarly, increasing whey protein substitution from 2.5% to 20% increased the protein content of bread from 12% to 32.85%, respectively. This substantial increase highlights whey protein's enrichment potential at high substitution levels, underscoring that preprocessing is a key factor in improving protein nutritional value (Delmi Bouras et al. 2022). Nevertheless, whey‐enriched products should not be evaluated by protein content alone. For example, the DIAAS value reported for whey‐fortified bread was markedly lower than that of whey protein alone, indicating that the farinaceous matrix and thermal processing may reduce the effective nutritional value of the incorporated protein. This discrepancy has been associated with the denaturation of whey proteins such as β‐lactoglobulin and α‐lactalbumin, which, after partial loss of tertiary structure, may interact with damaged starch in flour (Delmi Bouras et al. 2022). Damaged starch exposes hydroxyl groups that can bind to protein structures and contribute to the formation of thermally induced complexes, potentially reducing protein bioavailability (Gani et al. 2015). Therefore, although whey protein is generally regarded as a high‐quality animal protein, its effectiveness in baked systems depends strongly on matrix interactions, processing conditions, and the protein format used.

In this context, although cheesecake is not a farinaceous product per se, it serves as a useful comparative case to illustrate how protein enrichment behaves in a gel‐type matrix. MPC incorporated at an 84% replacement level yielded cheesecake with 25.5% protein and a 68.4% relative protein increase compared with the control. This ingredient demonstrated high potential for nutritional enrichment without compromising structural integrity. Its performance is related to the formation of a stable protein matrix through thermo‐acid coagulation and controlled denaturation of whey proteins, including β‐lactoglobulin and α‐lactalbumin, which helps prevent excessive water migration and syneresis (Deinychenko et al. 2020). However, this example also illustrates that high substitution levels may be feasible only in specific matrices, such as cheesecake, where the product structure naturally accommodates protein gelation. Therefore, the successful use of MPC in this case should not be directly extrapolated to bread, cookies, or pasta without considering matrix‐specific rheological requirements.

#### 3.1.2 Eggs and Egg Products

Powdered egg white has also been evaluated as a protein‐enriching ingredient in gluten‐free cookie formulations. At substitution levels of 15% and 30%, it produced a protein content of up to 14% and a relative protein increase of 323% (Sahagún and Gómez 2018). Despite this substantial improvement, its incorporation negatively affected dough rheology and final texture. This effect is mainly attributed to ovalbumin, which competes for water with other matrix components and alters water retention, water absorption index (WAI), and WBC (Cui et al. 2025). This case is particularly relevant because it demonstrates that large relative increases in protein content may be misleading when the control formulation has low initial protein levels or when enrichment compromises processing performance.

#### 3.1.3 Fish Powder

Fish powders have been used as ingredients in farinaceous products. Desai et al. (2018) reported that 15% substitution with salmon fish powder (SFP) increased protein content to 20% in bread, corresponding to a 44% increase relative to the bread control. Beyond protein enrichment, SFP conferred additional functional benefits, including partial inhibition of starch digestion, reduced glycemic index, and enhanced antioxidant capacity in the final product (Desai et al. 2018). These findings suggest that SFP may be useful not only for protein fortification but also for the reformulation of high‐glycemic starch‐based products. Nevertheless, future applications should evaluate sensory acceptance, lipid oxidation, odor stability, and consumer perception, since fish‐derived ingredients may introduce technological and sensory constraints that limit their practical incorporation into farinaceous products.

The combination of fish powder and whey protein in bread illustrates animal protein complementation. Fish powder contributes lysine, methionine, and cysteine, whereas whey protein provides threonine, leucine, isoleucine, and valine. Their combined incorporation achieved a biological value of 96.76% and met the required levels for most EAAs according to FAO/WHO recommendations for children aged 1–2 years, with lysine remaining the limiting amino acid but not deficient (Klyuchko et al. 2022). This formulation is relevant because it illustrates that animal‐derived proteins can also benefit from strategic blending, rather than relying on a single high‐quality source. In addition to nutritional complementation, whey proteins such as β‐lactoglobulin may contribute emulsifying properties that improve matrix stability, while fish proteins, particularly myosin, can enhance elasticity (Klyuchko et al. 2022). Therefore, the fish–whey combination demonstrates synergistic potential at both nutritional and technological levels, although sensory acceptance and lipid oxidation stability should be considered before broader application.

#### 3.1.4 Edible Insects

Edible insects are another source of animal protein used as an ingredient in farinaceous food formulations. These alternative proteins can significantly enrich these food matrices in protein and EAA content. Their protein content can be comparable to that of dairy ingredients such as whey and superior to that of cereal or grain proteins, although their EAA adequacy, particularly for lysine, is more variable. The main sources of edible insects are *Acheta domesticus* cricket, *Tenebrio molitor* mealworm, grasshopper (*Orthoptera* spp.), and cinereous cockroach (*Nauphoeta cinerea*) (Bottle et al. 2024; Haber et al. 2019; De Oliveira et al. 2017) (Table 4). Depending on the level of substitution of these flours in food formulations, the protein enrichment levels in the final product can range from 25.4% to 27.7% for a 10%–30% substitution of cricket flour, and between 14.8% and 18.1% for a 10%–20% substitution of grasshopper meal, reaching 22.7% with a 10% substitution of cinereous cockroach meal (Table 3). This shows that the insect type affects how much meal must be substituted to achieve similar enrichment levels.

As shown in Table 4, replacing 10% of wheat flour with flours derived from *A. domesticus* and *T. molitor* increased the protein content of the resulting matrices to approximately 19%. Among these ingredients, defatted *A. domesticus* flour showed the most favorable performance, reaching 20% crude protein and a 31% relative protein increase (Bottle et al. 2024). This improvement is partly explained by lipid removal, which can reduce protein encapsulation and lipoprotein complex formation, thereby improving digestive enzyme accessibility. In addition, the arginine‐ and threonine‐rich composition of *A. domesticus* flour may promote hydrogen bonding with wheat proteins and starch, because polar groups (amide, hydroxyl, and carboxyl) can interact with starch hydroxyl groups through noncovalent forces during dough mixing, contributing to network stabilization (Zhang et al. 2026; Hu et al. 2026). Therefore, defatting appears to be a relevant processing strategy not only to increase the relative protein fraction but also to improve protein accessibility and technological compatibility.

Similar improvements have been reported with other insect ingredients. A 20% replacement with defatted grasshopper powder increased bread protein content to 18.1% while preserving sensory and textural properties (Haber et al. 2019). Likewise, partial wheat flour substitution with cricket powder or cricket sourdough at 10%–30% produced one of the highest protein contents reported among the reviewed matrices, reaching 27.72% (Osimani et al. 2018). In addition, 10% incorporation of *N. cinerea* flour produced a 134% relative protein increase, reaching a final protein value of 22.7% (De Oliveira et al. 2017). This substantial improvement is associated with the high intrinsic protein content of *N. cinerea* and the efficiency of milling and sieving, which remove part of the fibrous exoskeleton and concentrate the protein fraction. Insect proteins such as actin and myosin may also contribute to the formation of structural networks that interact with starch and gluten during baking, potentially supporting matrix stability (Scholliers et al. 2019; Osimani et al. 2018). Nevertheless, the role of chitin remains ambivalent: while it may contribute to fiber enrichment and structural effects, it can also reduce protein digestibility by limiting enzyme access to the protein fraction.

As discussed for other animal‐based sources, insect‐based formulations showed high protein content but more variable protein quality. Despite their protein density, none of the insect matrices evaluated in Table 1 met the FAO's minimum lysine recommendation, indicating that insect proteins should not be assumed to be nutritionally complete in cereal‐based products (Rumpold and Schlüter 2013). In the case of the *T. molitor* full‐fat meal, which contained 17.5% protein and showed 86% digestibility, this value was slightly lower than that of the wheat bread control. This reduction has been attributed to chitin, an indigestible polysaccharide found in the exoskeleton that can restrict digestive enzymes’ access to protein fractions. The PDCAAS value of 62% reflects the combined effects of an incomplete EAA profile and reduced digestibility (Bottle et al. 2024). Processing strategies such as defatting, fine milling, fermentation, chitin reduction, the use of natural deep eutectic solvents, and microwave‐assisted extraction may improve protein accessibility by disrupting chitin–protein–mineral complexes (Mersmann et al. 2025; Haghayeghi et al. 2025).

In addition to increasing protein content and contributing to a more complete profile of EAAs, insect meal can interact with the food matrix and processing conditions. This influences dough rheology, leavening capacity, product volume, texture, color, and sensory properties. For instance, ingredients derived from ground crickets can negatively impact dough rheology, fermentation, and product volume. However, appropriate incorporation levels can keep these effects within acceptable technological and sensory limits. Levels of around 8%–10% typically provide an adequate balance between fortification and product quality (Osimani et al. 2018; Haghayeghi et al. 2025; Tilami et al. 2025). Conversely, bread formulated with 5% *T. molitor* flour had the highest specific volume (4.02 cm^3^/g), compared to breads made with alternative proteins such as *A. domesticus* (3.49 cm^3^/g) (Osimani et al. 2018), *Schistocerca gregaria* (1.98 cm^3^/g) (Haber et al. 2019), and *Nauphoeta cinerea* (3.06 cm^3^/g) (De Oliveira et al. 2017). The ability of *T. molitor* flour to maintain loaf volume is attributed to insect proteins with foaming properties that reinforce the continuous phase without compromising the gluten network (Bottle et al. 2024).

The reviewed studies indicate that animal‐derived proteins, including insects, can effectively improve the protein content of farinaceous products, with final protein levels reaching approximately 18%–33% under specific formulation conditions. However, performance depends heavily on ingredient format, substitution level, processing history, and compatibility with the target matrix. Concentrated, isolated, and hydrolyzed ingredients generally provide greater enrichment than unprocessed protein powders, but they may involve higher processing costs, energy demand, and sustainability concerns (Gao et al. 2020). More importantly, the evidence shows that crude protein content alone is insufficient to determine the success of animal protein fortification.

### 3.2 Plant‐Based Protein

Current trends in food innovation have promoted plant‐based protein sources, particularly legumes, pseudocereals, and oilseeds, as viable alternatives to animal proteins in farinaceous products. This growing interest in plant‐derived ingredients has led to extensive research on alternative flours, such as chickpea, buckwheat, and carob, for their ability to modify dough rheology and enhance the functional properties of farinaceous bakery products (Cingöz and Yörükoğlu 2022).

However, the nutritional quality achieved with these ingredients does not always match that of animal‐derived proteins, especially in terms of EAA balance and digestibility. Therefore, many plant proteins require complementary blending, targeted processing, or both, to obtain a more complete amino acid profile and improve their technological performance in bakery systems. Table 3 summarizes plant‐based protein sources incorporated into cereal‐based products, including whole, defatted, germinated, toasted, debittered, concentrated, and isolated forms.

#### 3.2.1 Soy (*Glycine max*)

Soy protein is one of the most widely used plant‐based ingredients in the food industry due to its high protein quality, high digestibility, and well‐balanced profile of EAAs. Its amino acid composition complements that of cereal proteins, particularly by providing lysine, which is typically limiting in wheat‐based products (Messina 2022). Soy meal contains approximately 5.51–6.26 g of lysine per 100 g of crude protein (Dozier and Hess 2011), whereas wheat protein contains approximately 2.7 g of lysine per 100 g of protein, which is below the adult reference pattern of 4.5 g lysine/100 g protein (45 mg/g) (FAO 2013). Defatted soybean flour and soy protein isolates (SPIs) contain substantially higher lysine concentrations than cereal grains, making them effective ingredients for improving the nutritional quality of cereal‐based foods (Acevedo‐Pacheco and Serna‐Saldívar 2016). In bakery applications, incorporating defatted soy flours (∼49% protein) and soy protein concentrates (∼67% protein) into wheat flour formulations increased bread protein content by about 23% and nearly doubled its lysine content compared with wheat flour controls.

Beyond their nutritional value, differences in the functional properties of soy ingredients significantly affected dough characteristics and bread quality, highlighting the importance of ingredient selection to optimize both the nutritional and technological properties of bakery products (Lazo‐Vélez et al. 2015; Lazo‐Velez et al. 2017). Soybean proteins exhibit functional properties such as water absorption and solubility indices (WAI and WSI), as well as protein dispersibility and nitrogen solubility indices (PDI and NSI), which influence dough rheology and final product quality. Moreover, combining soybean‐derived ingredients with other plant‐based ingredients can also improve technological performance. In bread, incorporating Qulle variety cassava flour (10%) and Clark 63k soybean flour (19%) resulted in a crude protein content of 18% (Ayele et al. 2017). This formulation is relevant because it illustrates how plant proteins and starch‐rich ingredients can interact to support both nutritional enrichment and technological functionality. During baking, soybean proteins, mainly glycinin and β‐conglycinin, undergo denaturation, exposing thiol groups that may participate in disulfide bond formation with wheat gluten. Meanwhile, cassava starch, rich in amylopectin, acts as a relatively inert filler that can modulate gluten network density. These interactions help maintain bread structure while improving protein content.

In terms of health benefits, proteins from different sources may confer several health benefits, many attributable to the generation of bioactive peptides during processing and gastrointestinal digestion. These peptides can exhibit diverse biological activities, potentially contributing to improved consumer health and well‐being. Soy proteins have also been associated with several health benefits, including improvements in blood lipid profiles, promotion of lean body mass, and the generation of bioactive peptides during digestion (Singh et al. 2014). These peptides have been reported to exhibit antioxidant, antihypertensive, hypocholesterolemic, antidiabetic, and antiobesity activities (Acevedo‐Pacheco and Serna‐Saldívar 2016). In addition, bioactive compounds naturally associated with the soybean matrix, particularly isoflavones, may further enhance functional value because of their phenolic structure, antioxidant potential, and relative stability during baking. However, although phytate hydrolysis during baking may improve mineral bioavailability, this effect must be interpreted cautiously, since phytate degradation depends on formulation, moisture, pH, and thermal conditions (Ayele et al. 2017).

#### 3.2.2 Lupinus (*Lupinus* spp.) and Sacha Inchi (*Plukenetia volubilis*)

Lupinus (*Lupinus* spp.) also represents a relevant plant protein source, although its performance depends on the species used and the processing treatment applied (Kłobukowski et al. 2021). When combined with Sacha inchi (*P. volubilis*) concentrates, lupinus‐based formulations showed relative protein increases ranging from 36% to 51%, with final protein contents of 20%–22% in bread (Álvarez‐Quinteros et al. 2025). More importantly, these formulations demonstrate the value of optimization‐based blending. Álvarez‐Quinteros et al. (2025) showed that balancing different protein matrices through an optimization algorithm can maximize EAA content while maintaining favorable sensory properties. This optimization is particularly relevant because lupin contributes high levels of lysine and phenylalanine, whereas Sacha inchi provides tryptophan, resulting in a more balanced EAA profile. Beyond this nutritional complementarity, lupin is rich in albumins and globulins, which may contribute to water absorption and dough structure.

According to Álvarez‐Quinteros et al. (2025), both matrices can be optimized to approach FAO‐recommended EAA values, although lysine remained slightly below the reference level. Specifically, the optimized formulation containing 4% lupin and 9% sacha inchi concentrates reached an AAS of 0.65, compared with 0.45 for the wheat control bread. Importantly, this formulation showed high protein digestibility (86%), likely because its tertiary structures are less compact than wheat prolamins, facilitating access by proteolytic enzymes such as pepsin, trypsin, and chymotrypsin. However, its PDCAAS of 0.56 indicates that high digestibility alone is insufficient when the AAS remains constrained by a limiting amino acid. This value was still higher than the control bread's PDCAAS of 0.40, confirming a net improvement in protein quality. This makes the formulation promising but also shows that lysine complementation remains necessary. Similarly, Cordero‐Clavijo et al. (2025) reported that substituting 6% of wheat flour with alkaline Sacha inchi protein extracts increased protein content by up to 26.4% and enhanced several EAAs, including leucine, threonine, tryptophan, valine, and BCAAs. Nevertheless, lysine remained the first limiting amino acid, and the authors recommended combining Sacha inchi with a lysine‐rich matrix such as soybean or lupin.

Along with their nutritional and technological advantages, the enriched breads showed selective in vitro cytotoxicity against Caco‐2 and MCF‐7 cancer cell lines, with IC_50_ values ranging from 5.4 to 6.1 mg/mL, while maintaining high viability in noncancerous HDFa cells (Álvarez‐Quinteros et al. 2025). This bioactivity has been associated with peptides and phytochemicals with antioxidant, anti‐inflammatory, and pro‐apoptotic properties, potentially enhanced by the optimized combination of lupinus and sacha inchi Cordero‐Clavijo et al. (2021). However, future work should clarify whether these bioactive effects are retained after digestion and whether they are attributable to specific peptides, phenolics, or synergistic interactions.

#### 3.2.3 Chickpea (*Cicer arietinum*) and Cowpea (*Vigna unguiculata*)

Incorporating chickpea (*C. arietinum*) and cowpea (*V. unguiculata*) flours and proteins into bakery formulations has been proposed as an effective strategy to improve the nutritional quality of cereal‐based products. Unlike oilseed legumes, these pulses are starch‐rich and combine high levels of protein (approximately 20%–25%), dietary fiber, and resistant starch, making them particularly suitable for bakery applications. Their protein content is reported to be two to four times higher than that of most cereal and tuber crops, while also exhibiting higher biological value and digestibility (Jayathilake et al. 2018). Although chickpea and cowpea proteins are relatively deficient in sulfur‐containing amino acids, they provide more lysine and arginine than cereal proteins, complementing cereal amino acid profiles and improving the overall protein quality of bakery formulations (Lazo Vélez et al. 2023).

#### 3.2.4 Pseudocereals: Amaranto (*Amaranthus hypochondriacus*) and Quinoa (*Chenopodium quinoa*)

The incorporation of pseudocereals such as quinoa and amaranth into farinaceous products can contribute to a more balanced EAA profile compared with conventional cereal‐based formulations. Incorporating *A. hypochondriacus* flour at 25% substitution in bread increased protein content to 18%, indicating adequate performance as a protein‐enriching ingredient (Miranda‐Ramos et al. 2019). Regarding quinoa, a protein isolate used as the primary egg substitute in cakes showed the highest protein quality among the evaluated plant matrices (Mohamed et al. 2025). Despite its relatively low total protein content of 7.90%, it met FAO recommendations for all EAAs except leucine and phenylalanine + tyrosine in children aged 1–2 years. This finding is relevant for egg‐free bakery applications, where quinoa protein may simultaneously improve amino acid quality and provide technofunctional value. Although egg white has superior foaming capacity, quinoa protein exhibits greater foam stability, which is critical for gas retention in dough and batter systems (Mohamed et al. 2025). This example also emphasizes that a lower‐protein formulation may present higher nutritional quality if its EAA balance and functional performance are favorable.

By contrast, formulations prepared with conventional quinoa flour showed both low protein content and incomplete EAA profiles (Peñalver and Nieto 2024). This difference highlights the importance of ingredient format. Unprocessed quinoa flour, unlike quinoa protein isolate, lacks sufficient protein density to substantially improve EAA adequacy. Therefore, selecting isolates, concentrates, or optimized blends is often necessary when the goal is to improve protein quality rather than simply increase protein content. Another relevant plant‐based formulation combined brown rice flour with encapsulated spirulina, meeting all EAA requirements and exceeding FAO recommendations for lysine at 5.81 g amino acid/100 g protein (Peñalver and Nieto 2024). This nutritional advantage stems from brown rice's composition, which, unlike quinoa, contains significantly lower levels of phytates and tannins and is completely devoid of saponins, ANFs known to compromise EAA bioavailability. Notably, brown rice is one of the most complete plant‐based protein sources in terms of EAAs, as highlighted by Cordero‐Clavijo, Espinosa‐Ramírez, et al. (2025). Nevertheless, its low total protein contribution within the final matrix may limit its practical relevance as a dietary protein source.

#### 3.2.5 Other Cereal and Leguminous Sources

Among the most complete plant‐based combinations, the composite of faba bean flour, carob bean flour, and gluten showed notable nutritional performance (Hoehnel et al. 2020). Faba bean flour is rich in lysine and arginine but limited in sulfur‐containing amino acids; carob bean flour contributes threonine and tryptophan; and gluten provides structural functionality, although it is poor in lysine. This formulation achieved the highest protein content among the analyzed plant‐based matrices, reaching 25%, and met FAO recommendations for all EAAs except lysine. Its PDCAAS of 77% indicates a comparatively high‐quality plant‐enriched matrix. Moreover, Hoehnel et al. (2020) reported superior in vitro and in vivo digestibility compared with conventional wheat bread, including 69% greater nitrogen utilization and a protein efficiency ratio of 2.13 versus 1.13 for standard bread. Nevertheless, because lysine remained limiting, supplementation with lysine‐rich ingredients, such as quinoa protein isolate, could further improve the biological value of the final product. This hypothesis should be tested through amino acid profiling, digestibility assays, ANF analysis, and sensory evaluation.

Among the bread matrices evaluated, the combination of faba bean flour (*Vicia faba*) (10%), carob bean flour (5%), and gluten (2.5%) showed particularly attractive results, reaching a protein content of 25% (Hoehnel et al. 2020). This formulation also exhibited antioxidant potential associated with flavonoids and tannins, compounds commonly reported in carob‐derived ingredients (Mekhoukhe et al. 2019; Fidan et al. 2020). These compounds have been related to reductions in lipid peroxidation and hydrogen peroxide levels (Elbouzidi et al. 2023) and may contribute to protective effects against oxidative‐stress‐associated chronic conditions, including cardiovascular diseases, hypertension, atherosclerosis, and metabolic disorders (Oluwole et al. 2022). Nevertheless, such biological effects should be considered as potential functional attributes rather than direct health claims for the final product, unless supported by bioaccessibility, in vivo, or clinical evidence. From a formulation perspective, this blend is relevant because it combines high protein content, complementary plant components, and preserved sensory quality, positioning it as one of the most promising plant‐based alternatives reported in the reviewed literature.

Additionally, in a recent review on colored cereal proteins, Cordero‐Clavijo, Espinosa‐Ramírez, et al. (2025) highlighted that proteins from colored cereals are emerging ingredients of growing interest because of their nutritional value and functional versatility. These proteins comprise different fractions, including albumins, globulins, prolamins, and glutelins, each exhibiting distinct nutritional, functional, and technological properties. The authors also emphasized their potential for the production of protein concentrates, isolates, and hydrolysates, as well as the role of conventional and emerging extraction technologies, such as ultrasound‐ and microwave‐assisted extraction, in enhancing their applicability. As a result, proteins from colored varieties of wheat (*Triticum* spp.), maize (*Zea mays* L.), rice (*Oryza sativa* L.), oat (*Avena sativa* L.), barley (*Hordeum vulgare* L.), and sorghum (*Sorghum bicolor* (L.) *Moench*) have been recognized as promising resources for the development of innovative farinaceous products and other health‐oriented food applications.

#### 3.2.6 Microalgae

Microalgae have emerged as innovative protein sources for food applications more broadly, with several species valued for their high protein content, typically ranging from 40% to 70% on a dry weight basis, including *Dunaliella bardawil*, *Chlamydomonas reinhardtii*, *Chlorella protothecoides*, *Chlorella vulgaris*, *Arthrospira platensis* (*Spirulina*), and *Euglena gracilis* (Prates 2025). Among these, *Spirulina*, *C. vulgaris*, and *Tetraselmis chuii* have shown strong incorporation potential in farinaceous food matrices, with documented effects on protein enrichment and technological performance (Table 4). Moreover, the incorporation and comparison of 4% *C. vulgaris*, *T. chuii*, and *P. tricornutum* microalgae significantly enhanced the protein content of wheat bread across all three species tested, with *C. vulgaris* demonstrating the most substantial relative protein increase of 14.7%, raising bread protein from 10.62 to 12.18 g/100 g (Mahmoud et al. 2024).

*Spirulina*‐ and *Chlorella*‐fortified products have been shown to be rich in EAAs, with *Spirulina* crackers being particularly rich in alanine, aspartate, and tryptophan, and *Chlorella* grissini stand out for higher contents of isoleucine, leucine, lysine, and valine, and showed high bioaccessibility after in vitro digestion (64% for grissini, 93% for crackers) (Hernández‐López, Abadias, et al. 2023). Furthermore, W. M. Qazi et al. (2021) highlighted that compared to wheat, *N. gaditana*, *T. chuii*, and *C. vulgaris* contain higher amounts of EAAs, and their incorporation has been shown to markedly improve protein quality in bread (DIAAS 57%–66% vs. 46%). However, the same authors mentioned that these fortified breads containing ethanol‐pretreated microalgae from these species have also demonstrated a notably reduced in vitro protein digestibility compared to the control wheat bread (51.1%–57.3% vs. 68.8%), primarily due to decreased protein solubility during simulated digestion rather than impaired enzymatic degradation of solubilized proteins. Still, despite the reduced in vitro protein digestibility observed in the fortified breads, the microalgae‐enriched breads still achieved good DIAAS values that substantially improved upon the control wheat bread, demonstrating that the higher EAA content and improved amino acid composition of the microalgae compensated for the digestibility limitations, making them effective for protein fortification purposes.

Additionally, enrichment with 1% and 2% *Spirulina* maintained EAA concentrations after baking, with total EAA reaching 48,209 and 55,286 mg/kg dry weight, respectively, demonstrating nutrient retention through thermal processing (Montevecchi et al. 2022). Moreover, considering lysine as the limiting amino acid in wheat‐based products, Wang et al. (2025) highlighted that 6% *Spirulina* addition in steamed buns generated a pronounced improvement in lysine content (79.72%) and total EAAs (57.53%) compared to control steamed buns, also mentioning that amino acids in *Spirulina* were available in both free form and as small peptides, which facilitates their digestion and absorption by the human body. Likewise, homemade biscuits fortified with 2% *A. platensis* demonstrated a rich amino acid profile with a 15%–18% total amino acid content increment and significant increases in EAAs, particularly histidine (threefold increase) and arginine, which are critical for infant and children's nutrition (Şahin 2019).

For gluten‐free bread applications, microalgae, such as *T. chuii*, at a 4% incorporation level, have been investigated for nutritional quality improvements due to their 47.7% protein content in dry weight, as they successfully recover dough structure and significantly increase antioxidant activity (Nunes et al. 2020). Moreover, incorporating *N. gaditana*, *C. vulgaris*, and *T. chuii* microalgae as enrichment significantly improves protein content and mineral composition, where *N. gaditana*’s EAA index score of 1.02 was found to be higher compared to the approved and GRAS (Generally Recognized As Safe) species like *Spirulina* (EAAI = 0.81) and *C. vulgaris* (EAAI = 0.92) (M. W. Qazi et al. 2022). At this point, it is also important to highlight the higher mineral content of the samples, which may be associated with the marine origin of the microalgal species. The mineral composition in the resulting enriched products should be an important issue to consider, as it can both positively and negatively affect protein digestibility and amino acid/peptide release through distinct molecular mechanisms. Positive effects occur when minerals facilitate proteolytic enzyme activity and protein unfolding during gastrointestinal digestion, thereby enhancing peptide‐bond accessibility and hydrolysis and increasing free amino acid and peptide release (Boskovic Cabrol et al. 2022). Conversely, high ash concentrations can form mineral–protein complexes or chelation interactions that reduce substrate accessibility to digestive proteases, thereby inhibiting enzymatic hydrolysis and limiting peptide and amino acid liberation (Thongthung et al. 2024). Therefore, the specific mineral type and ash concentration of the microalgae‐fortified products should be a key factor to consider, as different minerals exhibit varying affinities for protein binding sites and ion composition directly influences the molecular weight distribution and bioaccessibility of resulting peptides (Wahjuningsih et al. 2025).

Additionally, nutritional analyses reveal species‐dependent digestibility, ranging from 70% to 90%, with *A. platensis* achieving the highest rates due to low cellulose content in its cell wall (García‐Encinas et al. 2025). Research comparing different microalgae species confirms that *Chlorella* sp. and *A. platensis* achieve distinct digestibility coefficients, with cellular disruption through processing substantially improving protein bioavailability (Niccolai, Chini Zittelli, et al. 2019). Thus, processing methods including enzymatic treatment, cell wall disruption, and innovative drying could enhance protein accessibility and digestibility, establishing microalgae as sustainable, high‐quality alternatives to animal and plant‐based protein sources (Prates 2025).

The combination of pseudocereals and cereals with encapsulated spirulina also represents a promising strategy within emerging protein enrichment. Formulations based on amaranth, brown rice, and quinoa combined with encapsulated spirulina showed high relative protein increases of approximately 83% (Peñalver and Nieto 2024). In these systems, the complementary EAA profiles of pseudocereals and spirulina, together with sourdough fermentation, may contribute to improved protein bioavailability. Encapsulation is particularly relevant because it helps preserve spirulina‐derived bioactive compounds during processing, resulting in increased antioxidant capacity and phenolic compound content in the final products. This approach illustrates the importance of combining ingredient selection with protective technologies when working with bioactive‐rich protein sources.

In the case of microalgae, their supplementation presents complex technofunctional challenges affecting both conventional and gluten‐free applications (Atubukha et al. 2026; García‐Encinas et al. 2025). Microalgae addition influences the physicochemical and rheological characteristics of dough and resulting products, although the specific response varies significantly by application type and concentration. Specific examples include the application of *A. platensis* in pasta to enhance nutritional value and improve technological and biological properties (Koli et al. 2022). In crackers and grissini, incorporation levels of 1.5%–3.5% for *Spirulina*, *Chlorella*, and *Tetraselmis* have shown increased nutritional values while maintaining acceptable sensory attributes (Hernández‐López, Abadias, et al. 2023). Also, *Spirulina* powder has been successfully incorporated at 1.5% and 2.5% levels into different wheat flours, with 2.5% concentration samples being well accepted by consumers and demonstrating increased antioxidant capacity at the same time (Hernández‐López, Abadias, et al. 2023).

Likewise, lactic acid bacteria fermentation of microalgae such as *C. reinhardtii* represents an effective pretreatment strategy that reduces negative impacts on dough fermentation performance and improves overall bread quality at substitution levels of 4%–8%, reducing inherent green color and volatile compounds (Jin et al. 2024). Furthermore, incorporating 2.5% *C. reinhardtii* powder in crackers and cookies showed optimal technofunctional performance for stretching energy and expansion through enhanced protein–gluten interactions (Yuan et al. 2025). At this substitution level, the products exhibited significantly higher stretching energy and stretch ratios attributed to *C. reinhardtii* proteins’ ability to facilitate cross‐linking or cohesion within gluten networks. Still, the hydrophilic properties of these proteins increased water absorption capacity from 57.2% to 59.10%, facilitating gel network formation and achieving the highest expansion ratios while reducing chromaticity values by inhibiting Maillard browning.

In gluten‐free formulations, *T. chuii* have demonstrated a dose‐dependent effect: at lower additions (1%–2%), it exhibits a destabilizing effect with reduced dough viscoelastic functions, while at higher levels (4%), structure recovery occurs with increased bread volume and decreased crumb firmness (Nunes et al. 2020). Moreover, adding *A. platensis* and *C. vulgaris* to wheat crackers at 2% resulted in stable color and texture throughout 8 weeks of storage, with gas retention not significantly affected, suggesting their suitability for applications requiring consistent product characteristics. However, at higher incorporation levels (6%), both species produced slightly thinner and lighter crackers, though density remained statistically constant (Batista et al. 2019). Furthermore, incorporation of microalgae biomass into gluten‐free bread has been shown to significantly compromise dough rheology and bread quality, with raw biomass (particularly *C. vulgaris* and *T. chuii*) reducing bread volume while increasing crumb firmness and dough softening. However, ethanol pretreatment of the biomass substantially improves these technofunctional properties by purifying algae proteins and eliminating pigments and volatile sulfur compounds. In this sense, ethanol‐pretreated *N. gaditana* biomass has been shown to strengthen dough structure, enabling greater air entrapment during fermentation, resulting in improved bread volume, reduced firmness, and superior sensory acceptance comparable to control bread (M. W. Qazi et al. 2022).

#### 3.2.7 Macroalgae (Seaweeds)

Seaweeds (macroalgae) are also gaining attention as low‐calorie, high‐protein, and low‐fat ingredients for farinaceous product enrichment. They could be an important source of protein and other nutrient compounds (hydrocolloids, minerals, vitamins, and bioactive compounds). The protein content of seaweed can range from 3% to 47%, depending on different macroalgal species. Red seaweeds generally exhibit higher dry‐protein (20%–47%) compared with brown (3%–15%) and green and (9%–26%) species; for example, *Porphyra* and *Pyropia* species (red seaweed) can reach around 47% (d.w.) protein, whereas *Adenocystis* (brown) and *Codium* (green) contain 12.6 g/100 g d.w. and 14.8 g/100 g d.w., respectively. In general, the protein content may vary seasonally depending on the availability and uptake of nutrients (N and P) (Gordalina et al. 2021; O’ Brien et al. 2022; Arakaki et al. 2023).

Most macroalgae contain all the EAAs required by humans, accounting for around half of the total amino acid content. These proteins are particularly rich in aspartic acid and glutamic acid residues. Some amino acids, such as threonine, lysine, tryptophan, and histidine, as well as sulfur amino acids (cysteine and methionine), are present in higher concentrations than in terrestrial plants. The amino acid profiles of certain macroalgae are like those of egg albumin and legumes. Among macroalgal species, *Chondrus crispus* demonstrated significantly superior amino acid profiles compared to *Palmaria palmata* and *Ascophyllum nodosum*, exhibiting notably higher concentrations of EAAs including leucine (28.16 ± 0.70 g/16 g N), lysine (17.28 ± 0.31 g/16 g N), and isoleucine (11.32 ± 0.24 g/16 g N), as well as prominent nonessential amino acids such as glutamic acid (51.75 ± 3.03 g/16 g N) and alanine (28.79 ± 0.31 g/16 g N), positioning it as a superior source of complete protein among some macroalgae species (Čmiková et al. 2024).

Besides this, comparing micro‐ and macroalgae protein digestibility, Khemiri et al. (2021) reported that microalgae‐fortified couscous formulations with honey and organic *Chlorella* demonstrated significantly higher in vitro protein digestibility compared to the mixture of *C. vulgaris* plus *Ulva rigida* and *Fucus vesiculosus*, which showed protein digestibility similar to the control sample without algae. This lower digestibility may be related to fucoidan, a sulfated polysaccharide from brown macroalgae, which can form protein–polysaccharide complexes that alter protein conformation, increase digestive fluid viscosity, and inhibit digestive enzyme activity (Fontes‐Candia et al. 2025). Fucoidan has also been shown to disrupt the gluten network in wheat‐based products by competing for water and breaking hydrogen bonds between gluten proteins (Yang et al. 2024); although that study assessed dough texture and starch digestibility rather than protein digestibility directly, it supports a plausible structural pathway by which fucoidan could also affect protein matrix integrity in farinaceous products. Together, these compositional and structural mechanisms indicate that macroalgae‐derived fucoidan could affect the protein fraction of farinaceous products, and this should be considered in the design of future macroalgae‐based formulations, warranting direct assessment of protein digestibility. Moreover, Terriente‐Palacios and Castellari (2022) showed that commercially available crackers and pasta formulated with algae ingredients contained high levels of the low‐molecular‐weight sulfonic acid derivatives taurine, hypotaurine, and homotaurine, indicating that these products can provide levels comparable to those found in foods of animal origin. Therefore, selection of suitable algae species and incorporation levels is critical to preserve bioactivity through food processing while maintaining consumer acceptance.

Given the bioactive properties of macroalgae, researchers have identified many bioactive peptides in the protein sequences of various macroalgae that show effects such as antihypertensive, antioxidant, anti‐inflammatory, and anticancer activities. However, the nutritional utilization of macroalgae poses a particular challenge compared to animal‐derived proteins such as casein and whey, which are more easily digested. This is because the digestion of algal proteins is greatly affected by phenolic compounds and other anti‐nutritional factors in the cell wall (polysaccharides), which restrict access to the proteins for gastrointestinal enzymes (Gordalina et al. 2021; O’ Brien et al. 2022).

Macroalgae have been incorporated into bread, pasta, noodles, and gluten‐free doughs, among other products (Table 4). *Ulva lactuca* and *Gelidium corneum* are examples of macroalgae that can be used in breadmaking. They have demonstrated significant protein enrichment potential, with bread formulations achieving protein increases of 12.1% and 14.4%, respectively, while maintaining excellent sensory properties (El Boujamaai et al. 2025). As well, *Saccharina latissima* flour at 1.5%–6% incorporation in bread maintained sensory properties similar to those of control samples at low levels (1.5%–3%), serving as sources of fiber and minerals while increasing the protein content by 26.2% (Oprea et al. 2023). Interestingly, this effect appears to be both matrix‐ and dose‐dependent. Tyl et al. (2024) demonstrated that adding fermented *S. latissima* or *Alaria esculenta* powders negatively impacted gluten protein aggregation in a dose‐dependent manner. The proteins from macroalgae likely contributed to the increased monomeric fraction, and algae particles appeared to restrict protein aggregation, limiting the size of gluten proteins. This mechanism weakened the dough matrix and reduced bread specific volume, where fermented *S. latissima* showed the most favorable sensory properties among the tested fermented macroalgae.

Still, several authors have mentioned that the incorporation of macroalgae could benefit the distribution of the proteins present in the elaborated farinaceous product rather than the protein content. This shift in protein distribution reflects a dilution of the gluten fraction that is not unique to macroalgae but has also been reported for other nonconventional ingredients, such as legume flours (Laleg et al. 2017), and more broadly across protein‐ and fiber‐rich enrichments including legumes, oilseeds, and microalgae (Mercier et al. 2016). Beyond bread, this dilution effect has also been documented in pasta. Ribeiro et al. (2022) showed that the incorporation of *F. vesiculosus* flour in pasta made the gluten proteins proportionally fewer, causing this dilution effect and a weakening of the pasta structure, underscoring the importance of considering not just protein quantity but also protein functionality and composition when formulating macroalgae‐fortified foods. At low inclusion levels (3%), *F. vesiculosus* enrichment produced favorable textural modifications with lower hardness alongside increased adhesiveness and resilience (Ainsa et al. 2022), while simultaneously requiring additional water during dough formation and extending cooking time (Ribeiro et al. 2022). Proportionally, at higher inclusion percentages (5.5%–10%), these effects intensify, with substantially increased cooking loss and more pronounced texture alterations becoming apparent (Ribeiro et al. 2022). In addition, color variations in enriched pasta are also relevant due to differences among seaweed species. *Saccharina latissima* flour at 1.5% maintained rheological properties similar to control samples, while at 3%–6% levels, rheological and sensory properties degraded in a dose‐dependent manner, with firmness and gumminess increasing with storage time (Oprea et al. 2023).

Furthermore, in the case of instant noodles, red seaweed *E. denticulatum* proteins and polysaccharides significantly enhanced cooking quality by increasing water absorption and cooking yield to 144.18% compared to 97.00% in control noodles, while also reducing cooking loss (Koh et al. 2022). However, these proteins negatively impacted texture by interfering with gluten–starch interactions, resulting in substantially weakened tensile strength and hardness across all seaweed formulations. Despite these textural compromises, *E. denticulatum* proved valuable as a functional ingredient, improving food safety by reducing pH and water activity while maintaining acceptable overall quality. Still, the water activity of seaweed‐enriched products emerges as a critical parameter influencing shelf‐life quality, with higher moisture content during storage directly correlating with increased cooking losses and reduced firmness in cooked products (Ribeiro et al. 2021).

The technofunctional changes observed when macroalgae were incorporated into the analyzed products demonstrate that, although this improves the products’ nutritional value and functional properties, careful optimization of the level of incorporation is essential to maintain acceptable culinary quality and ensure that consumers find the products acceptable in terms of their sensory properties (Ribeiro et al. 2022; Milde et al. 2022). While macroalgae generally have a lower and more variable protein content (3%–47% dry weight) than microalgae, red and green macroalgal species contain significant nonstarch polysaccharides such as alginate, carrageenan, and agar that are beneficial for structure and texture improvement in gluten‐free dough systems, making them valuable functional ingredients beyond their protein content contribution (Tavares et al. 2023; Demoor et al. 2025). Additionally, the higher mineral content of macroalgae, owing to their marine origin, should also be considered, as mineral–protein interactions may influence protein technofunctional properties, as previously discussed for microalgae. In addition, combining microalgae and macroalgae may be advantageous; Khemiri et al. (2021) showed that the combination of *C. vulgaris*, *U. rigida*, and *F. vesiculosus* is better suited for consumers prioritizing mineral enrichment and texture quality besides the protein content.

## 4 Optimizing Strategies for Protein Enrichment of Farinaceous Products

The protein enrichment of the farinaceous products impacts their nutritional, technological, and functional properties. Thus, careful optimization of the process is crucial (Figure 2). Regarding the supply of EAAs, which directly influences the biological value of food, several studies recommend balancing the amino acid profile by combining protein sources. Optimal mixing of different matrices (i.e., soybean, hemp, chickpea, faba bean, lupinus, sacha inchi, and pea) can lead to a more complete EAA profile, especially higher lysine indices (the first limiting amino acid in farinaceous and in cereal‐based products) (Cutroneo et al. 2024; Monnet et al. 2019). The amino acid complementation has been achieved through mixture design, optimization algorithms, and response surface methods. For example, Álvarez‐Quinteros et al. (2025) used a nonlinear GRG (generalized reduced gradient) algorithm to optimize the amino acid profile of breads enriched with Lupinus and Sacha inchi protein concentrate mixtures. The protein‐enriched breads met the FAO amino acid recommendations for children aged 1–2 years. The optimization was constrained to minimize amino acid waste and to avoid exceeding a substitution level that could negatively affect the rheology of the matrix (bread). Furthermore, the authors highlighted that this optimization system can also be applied to design protein blends with balanced amino acid profiles tailored for the nutritional needs of various population groups, such as athletes and older adults. In a similar scenario, Roncolini et al. (2019) investigated the balancing of the amino acid profile of wheat bread fortified with *T. molitor* flour. The authors optimized the EAA content by calculating the ratio between the AAS for each formulation (with substitution levels ranging from 5% to 15%) and the FAO/WHO reference patterns. Formulations up to 10% substitution succeeded in maintaining acceptable dough properties, loaf volume, and crumb texture. This study demonstrates that even insect protein can be effectively incorporated into farinaceous products, enhancing nutritional value without compromising technological or sensory quality.

**FIGURE 2:**
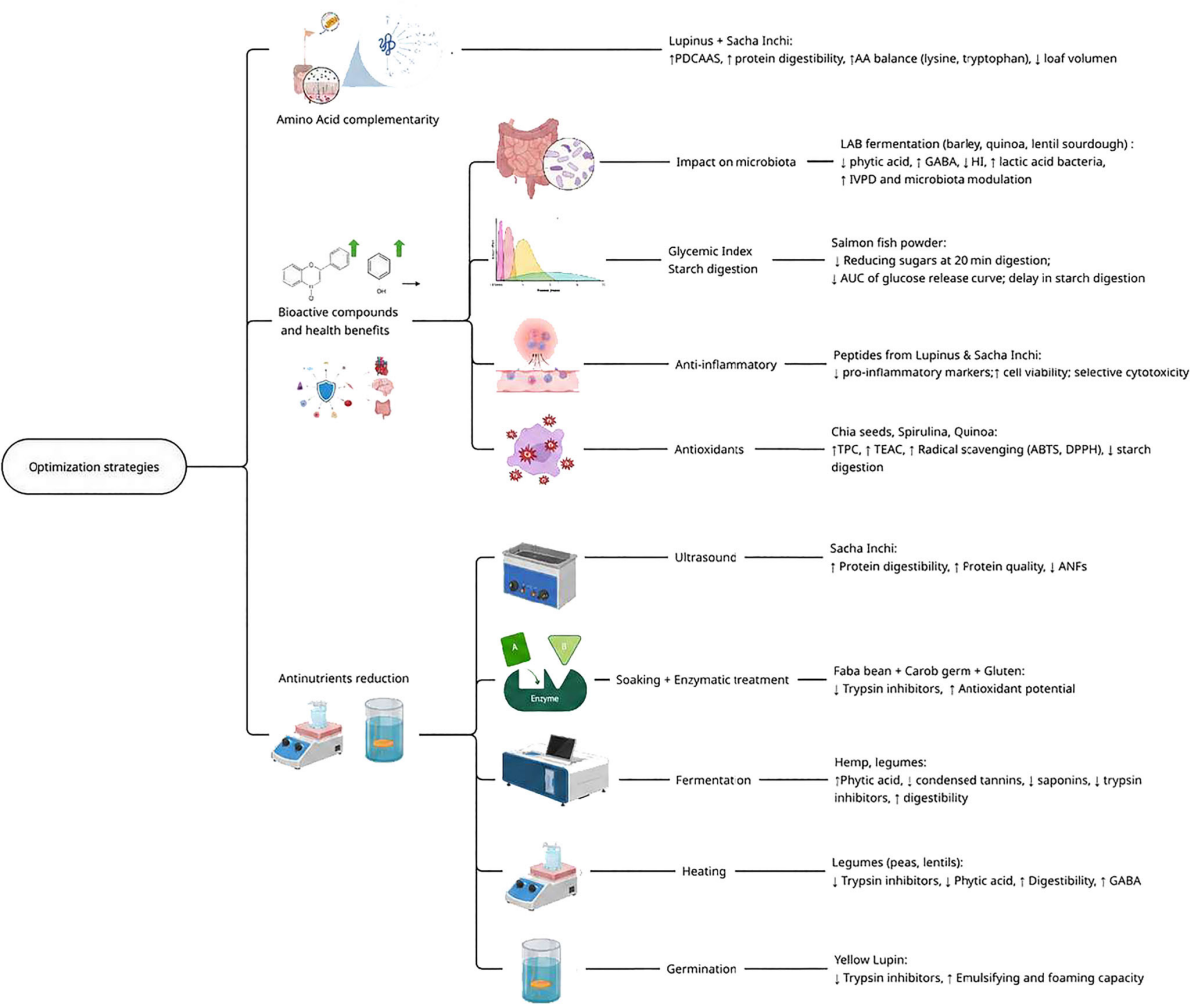
Strategies for improving protein quality, bioactive functionality, and antinutrient reduction in protein‐enriched farinaceous products.

**FIGURE 3:**
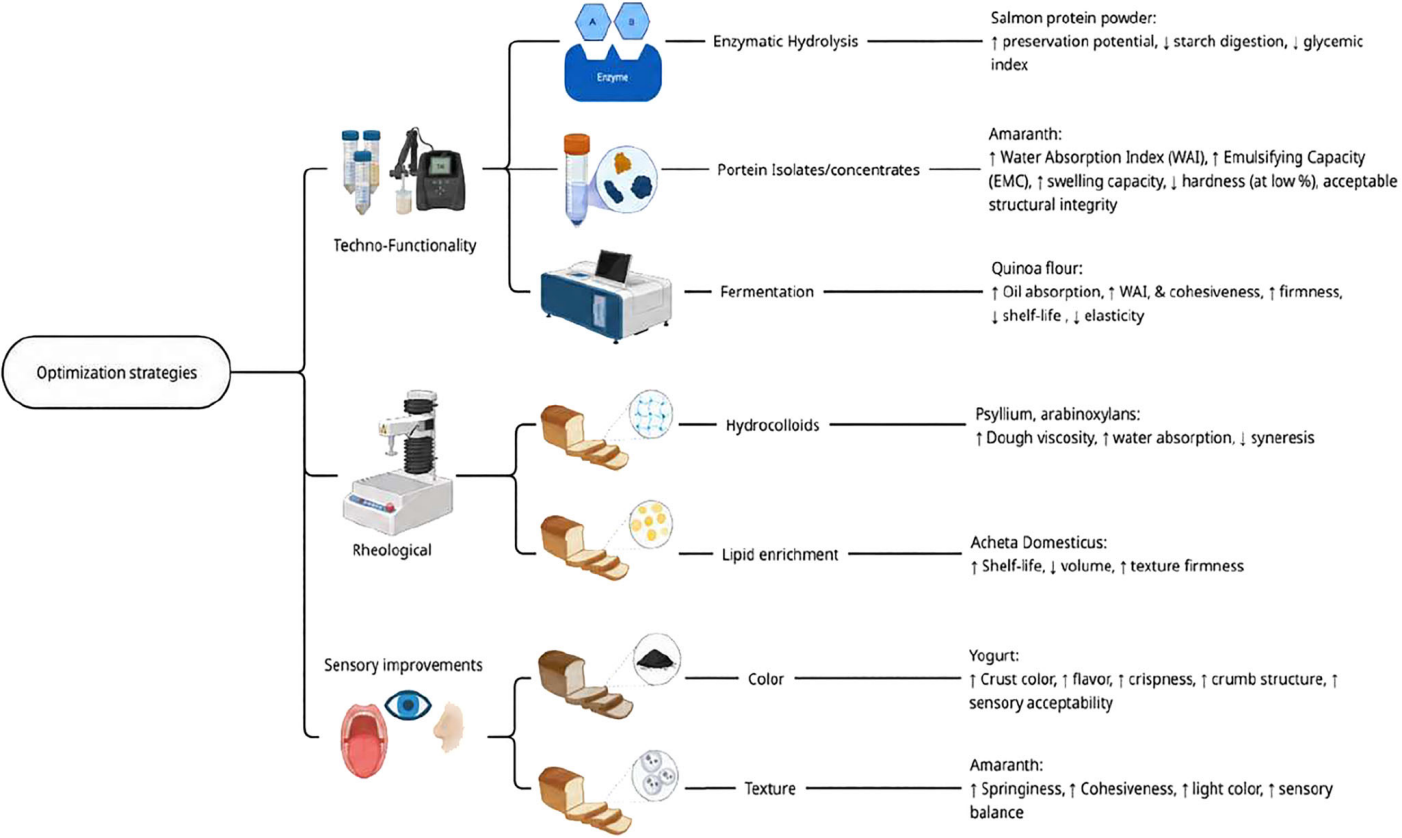
Technofunctional, rheological, and sensory optimization pathways in protein‐enriched farinaceous products.

More specifically, incorporating proteins into farinaceous foods involves complex protein–starch–fiber interactions that can create technological challenges related to texture, rheology, and sensory quality (Monnet et al. 2019). To overcome these limitations, strategies such as enzymatic hydrolysis and ultrasound‐assisted processing have been explored. In this context, enzymatic hydrolysis has been used to improve the solubility, emulsifying, foaming, and gelling properties of protein ingredients intended for farinaceous products. For instance, Knežević‐Jugović et al. (2023) produced soy protein hydrolysates using several commercial food‐grade proteases for incorporation into a cookie formulation. The combination of Neutrase and Flavourzyme yielded the most favorable results, with higher measured total EAA (by 9.8%) and lysine contents (by 32.6%) than the untreated soy protein control. These differences should be associated with protein breakdown and amino acid recovery or accessibility following enzymatic treatment. The resulting hydrolysates also maintained sensory acceptability, with consumption intention above 80%. Furthermore, their incorporation into wheat flour significantly reduced starch retrogradation, potentially extending cookie shelf life.

By contrast, Nemś et al. (2022) achieved a greater increase in total protein content through direct pea flour fortification, reaching 134.0 g/kg at 30% substitution compared with 66.9 g/Kg in the control, while correcting lysine limitation at substitution levels ≥20%. However, increasing pea incorporation progressively impaired sensory quality and increased product hardness, restricting its practical inclusion. Together, these findings suggest that enzymatic hydrolysis and direct fortification address different aspects of protein enrichment. Hydrolysis can improve protein functionality and accessibility, whereas direct fortification more strongly increases total protein content and may help correct amino acid limitations.

In another study, Shay et al. (2023) evaluated the impact of controlled enzymatic hydrolysis using alcalase on vegetable proteins from peas, rice, hemp, and oats. The goal was to improve the proteins’ solubility and surface properties for future use in farinaceous matrices. The study determined that the optimal hydrolysis conditions were similar for all matrices: 4% alcalase (g enzyme/g protein) at pH 8.0 and 50°C for 30 min. Following hydrolysis, the solubility of pea and oat proteins improved rapidly, and their surface hydrophobicity decreased significantly. Hemp protein hydrolysis produced larger polypeptides (∼20 kDa) and increased hydrophobicity, suggesting exposure of internal basic subunits without extensive cleavage. Conversely, rice protein showed a limited response to alcalase hydrolysis under these conditions. This work demonstrates that enzymatic hydrolysis improves solubility and modulates surface charge and exposure of key functional groups. These factors can favor the technological integration of plant proteins in farinaceous products by improving dispersion and structural compatibility.

In contrast, ultrasound‐assisted processing has emerged as a promising physical approach to enhance the incorporation of protein ingredients into farinaceous matrices. At the protein level, ultrasound can disrupt disulfide bonds and expose hydrophilic groups, thereby enhancing protein solubility, water absorption, and WBC (R. H. Majeed and Hashim 2024). Ultrasound treatment has also been associated with improved foaming properties, which may favor hydration behavior and contribute to softer protein‐containing matrices; however, the magnitude and technological relevance of these effects remain matrix‐dependent (Pi et al. 2025; Alrosan et al. 2026). Some studies demonstrate that ultrasonic treatment significantly reinforces the gluten network, improves dough handling, and enhances final product quality (Cao et al. 2022; Zhao et al. 2025). In surimi–wheat noodles, ultrasound‐assisted resting (optimal at ∼21 W/L) produced a denser, more continuous protein–starch network, reduced free sulfhydryl groups, improved water binding, lowered creep strain, and enhanced elasticity, chewiness, and cooked texture (Cao et al. 2022). Also, in steamed bread enriched with soybean and treated with ∼300‐W ultrasound, the hardness decreased by 18%, the specific volume increased by ∼15%, and structural analysis confirmed increased β‐sheet/random coil content and stronger gluten–protein interactions, along with reduced beany flavor components (∼7%–8%) (Zhao et al. 2025).

Another strategy for optimal technofunctional properties and rheology in protein‐enriched farinaceous is the use of protein isolates and concentrates. This is because of their good technofunctional properties, including high water and oil solubility, as well as emulsifying, foaming, and gelling capacities (Cordero‐Clavijo, Espinosa‐Ramírez, et al. 2025). Defatting, which concentrates protein in unrefined meals, can provide similar benefits. For example, Bottle et al. (2024) explored the impact of whole and defatted insect meals (*T. molitor* and *A. domesticus*) into bread formulations. The partial substitution of wheat flour with defatted meals (up to 10%) significantly increased water absorption in dough formulations. This enhanced hydration capacity improved dough workability and resulted in significantly lower hardness values during storage compared to control breads. This indicates a positive effect on crumb softness and texture retention over time. Further studies have explored integrating protein concentrates and isolates into farinaceous products, yielding promising results regarding the preservation of technofunctional properties. Neji et al. (2023) reviewed the impact of legume proteins, such as pea, soy, and lupinus, on dough systems. They found that using these proteins at moderate levels (≤10%) can enhance water absorption and viscoelastic modulus without substantially impairing dough stability or extensibility. Nogueira et al. (2022) compared the rheological properties of wheat dough enriched with pea protein isolate (PPI), SPI, and WPI. They observed that up to 10% substitution with PPI or SPI maintained acceptable water absorption and rheological behavior. However, WPI induced a more pronounced weakening of the gluten network. Regarding gluten‐free applications, incorporating 8%–12% whey protein concentrate (WPC) into sorghum and corn‐based doughs improves dough handling by modulating water absorption and reducing elastic modulus, resulting in softer, more cohesive structures (Zhu et al. 2023). Collectively, these findings underscore the potential of select protein sources to enhance the protein quality of farinaceous products without compromising their processing behavior or end‐product quality.

Additionally, some technological strategies have demonstrated promising results in reducing ANFs and enhancing the bioactivity of protein‐enriched farinaceous products. Espinosa‐Ramírez et al. (2022) found that using germinated legume flours effectively decreased trypsin inhibitor activity and improved protein digestibility in bread formulations enriched with *Phaseolus coccineus*. Similarly, Guardado‐Félix et al. (2020) found that adding sprouted chickpea flour, with or without selenium enrichment, significantly reduced antinutritional compounds and improved the nutritional quality of yeast‐leavened breads. This confirms the dual role of germination in enhancing nutrients and mitigating antinutrients. In both studies, germination resulted in a significant decrease in trypsin inhibitor activity, which is attributed to the activation of endogenous proteases during germination. Similarly, legumes can activate several endogenous enzymes, such as proteases, phytases, and amylases, during germination. These enzymes break down proteins, phytates, oligosaccharides, and other ANFs (Avilés‐Gaxiola et al. 2018).

Moreover, recent studies have demonstrated the potential of protein enrichment strategies to simultaneously deliver functional health benefits. For example, olive pomace protein incorporated into fermented bread has been shown to have significant anti‐inflammatory effects. These effects are attributed to the protein's high content of phenolic and bioactive compounds, which modulate inflammatory responses in intestinal cell models (Di Nunzio et al. 2020). Similarly, durum wheat pasta enriched with linseed flour demonstrated enhanced anti‐inflammatory and antihypertensive properties due to the synergistic effects of omega‐3 fatty acids, phenolic compounds, and a moderate increase in protein content (Amri et al. 2024). Legume protein hydrolysates, particularly those from faba beans and Lupinus, used in breads and snacks, have demonstrated antioxidant, antihypertensive (via ACE‐inhibitory peptides), and antiproliferative effects. This suggests their dual role as nutritional and functional ingredients (Tawalbeh et al. 2023). Additionally, incorporating concentrated broad bean pod protein extracts into snack‐type matrices has shown promise for anti‐inflammatory and anticancer activity in vitro, primarily due to protein‐bound polyphenols and saponins (Elbadrawy and Mostafa 2024). Thus, the potential for designing protein‐fortified starchy foods with specific functional properties can be achieved through strategic ingredient selection.

Incorporating microalgae into farinaceous products requires optimizing inclusion levels and pretreatment strategies to manage sensory challenges. For example, ethanol pretreatment of microalgae biomass effectively reduces green coloration and unpleasant odor compounds while preserving bread sensory properties, enabling higher substitution levels without compromising acceptability (W. M. Qazi et al. 2021). As discussed in Section 3.2.6, lactic acid fermentation of microalgal biomass can also reduce green color and off‐flavors while generating desirable aroma compounds (Jin et al. 2024; Nicolotti et al. 2025). Also, sourdough technology combined with microalgae incorporation at 2%–10% *Spirulina* in crostini products has been shown to provide health claims as a protein source while improving antioxidant capacity and phenolic content (Niccolai, Venturi, et al. 2019). Therefore, when incorporated into bread and other farinaceous products, microalgae can be added at various substitution levels, ranging from 1.5% to 12% of flour, depending on the desired nutritional outcome and sensory acceptability (Hernández‐López, Abadias, et al. 2023; W. M. Qazi et al. 2021); in this way, a combination of appropriate algae species selection and optimal incorporation levels ensures both nutritional enhancement and acceptable technological and sensory properties (Wang et al. 2025). For long‐term sustainability, microalgae cultivation on nonarable land and integration into circular biorefineries support environmental goals (Mosibo et al. 2024), while advances in extraction and purification techniques, including enzymatic hydrolysis and pulsed electric fields, maximize protein functionality and recovery efficiency (Ali et al. 2025), positioning microalgae as a viable eco‐efficient solution for sustainable protein fortification of farinaceous foods.

For macroalgae, optimization requires balancing the nutritional benefits with potential impacts on cooking properties and texture. The goal is to maximize the protein and bioactive compound contribution while maintaining acceptable sensory and technological attributes. In this sense, macroalgae proteins can be extracted from the algal biomass using methods that differ in how they disrupt the cell wall to release intracellular protein. Ultrasound‐assisted extraction works through acoustic cavitation, in which imploding cavitation bubbles release mechanical energy that physically ruptures the cell wall, while microwave‐assisted extraction converts microwave energy into heat via ionic conduction and dipole rotation, damaging the cell wall and membrane and promoting the release of intracellular proteins into the extraction solvent. Other approaches include solid–liquid extraction, based on solvent‐driven lysis like osmotic shock followed by alkaline extraction, and enzyme‐assisted extraction, which uses carbohydrases such as cellulases and hemicellulases to enzymatically break down the cell wall. Once extracted, seaweed proteins can be hydrolyzed, either chemically (using acids or alkali) or enzymatically (using proteases such as Alcalase, trypsin, or pepsin), into smaller peptide sequences to release bioactive peptides with potential antioxidant, anti‐inflammatory, antimicrobial, and antihypertensive activities (Echave et al. 2021).

## 5 Legal and Regulatory Perspectives on Microalgae and Insects

Alternative protein sources offer substantial environmental sustainability benefits, exhibiting reduced greenhouse gas emissions and lower land and water resource demands compared to conventional livestock production systems (Sutula and Ryabukhin 2024). Nevertheless, critical implementation barriers persist regarding large‐scale adoption, particularly consumer adoption challenges. Food neophobia and the perception of insects as disease vectors evoke rejection, particularly within Western cultures. This challenge is further exacerbated by unfavorable organoleptic attributes, including off flavors associated with lipid oxidation, gritty textures, and dark coloration, which markedly diminish consumer acceptance, especially in bread. Overcoming these barriers requires integrated strategies encompassing processing technologies, product reformulation, and consumer‐friendly communication to reshape consumer perceptions (Medeiros et al. 2024).

Additionally, the commercialization of insect‐derived proteins is constrained by a fragmented and stringent regulatory landscape. In the European Union, their mandatory classification as Novel Foods under Regulation (EU) 2015/2283 (European Commission 2015) entails lengthy and costly safety assessments for each species. Article 3(2)(a)(v) explicitly includes “whole insects and their parts” within its scope, requiring comprehensive evidence on safety, allergenicity, and traceability. This requirement is critical because insect proteins share conserved pan‐allergens such as tropomyosin and arginine kinase with crustaceans and house dust mites, and clinical sensitization rates of up to 21.76% have been reported in allergic cohorts, although only a minority develop clinical symptoms upon controlled challenge (Liu et al. 2026). Furthermore, Commission Implementing Regulation (EU) 2017/2470 (European Commission 2017) establishes the Union List of authorized novel foods, and each insect species must be individually listed following a positive safety assessment by the European Food Safety Authority (EFSA). This case‐by‐case authorization framework necessitates extensive toxicological, nutritional, and allergenicity data for each species, as illustrated by recent authorizations such as UV‐treated *T. molitor* powder under Commission Implementing Regulation (EU) 2025/89 (European Commission 2025). Collectively, these regulatory challenges deter investment, limit innovation, and delay the industrial scalability of this emerging sector (Medeiros et al. 2024).

For algae, specific maximum inclusion levels in food products are not standardized globally. In developed markets, the EU requires individual authorization for specific algae species through its Novel Food framework without establishing universal percentage limits (Mendes et al. 2022), while the United States allows certain species (such as *Arthrospira*, *Chlorella*, and *Schizochytrium*) to be used under GRAS status without specific percentage restrictions (Su et al. 2023). In Asia, which dominates global algae production, regulatory frameworks vary significantly by country and focus primarily on contaminant limits (heavy metals, microbial contamination) rather than maximum inclusion percentages (Su et al. 2023). This contaminant‐focused regulatory approach reflects genuine safety concerns documented in the literature: heavy metals and rare earth elements have been detected in the majority of commercial microalgae‐based food products analyzed, with carcinogenic risk estimates for arsenic, cadmium, and chromium exceeding established safety thresholds in a substantial proportion of cases (X. Wu et al. 2024), while microalgal toxins such as microcystins and anatoxin‐a are heat‐stable and can persist through conventional food processing methods including cooking and pasteurization, posing an ongoing risk even after thermal treatment of the final farinaceous product (Withana et al. 2025).

In developing regions, regulatory gaps are more pronounced: Latin America and the Caribbean lack specific algae legislation, with major economic integration organizations relying on general Codex Alimentarius regulations instead (Gómez Gómez et al. 2024). FAO (2025) has explicitly identified the need for clearer, more efficient, and coordinated regulatory frameworks for macroalgae in the region, noting that despite traditional consumption practices and rich marine ecosystems, Latin America accounts for only 2% of global seaweed production. In Africa, modern regulatory frameworks for algae‐based foods remain largely underdeveloped despite historical consumption practices (R. Majeed et al. 2025; WHO 2013). As a result, inclusion levels are determined case by case based on product type and intended use rather than regulatory maxima, making standardization a key challenge for the global algae food industry and for incorporating algae into farinaceous products.

## 6 Future Perspectives

Future research should prioritize formulation strategies that integrate nutritional optimization with technological feasibility. Mixture design, response surface methodology, and optimization algorithms can support the rational combination of protein ingredients to maximize EAA adequacy while minimizing amino acid excess, sensory defects, and structural disruption. Likewise, processing strategies such as fermentation, germination, enzymatic hydrolysis, ultrasound, microencapsulation, defatting, and cell wall disruption may improve digestibility, reduce ANFs, preserve bioactive compounds, and enhance protein–matrix compatibility. However, these approaches should be evaluated not only in terms of protein enrichment but also through standardized digestibility assays, postdigestion bioaccessibility, storage stability, sensory acceptance, and, when possible, physiological validation.

Looking ahead, emerging technologies may further expand the protein toolbox available for farinaceous product innovation. Precision fermentation and molecular farming offer opportunities to produce proteins with tailored amino acid profiles, improved functionality, and potentially lower environmental impact. Nevertheless, these technologies will require rigorous assessment of scalability, safety, regulatory acceptance, consumer perception, and compatibility with food matrices before broad implementation.

## 7 Conclusions

A comprehensive assessment of protein enrichment in farinaceous products should move beyond crude protein content and adopt an integrated protein‐quality framework. In this context, the evaluation of animal, plant‐based, and emerging alternative proteins should include EAA profile, AAS, limiting amino acid, digestibility, biological value, and, when possible, standardized indices such as PDCAAS and DIAAS. These parameters are essential to determine whether protein fortification results in a true nutritional advantage rather than a merely quantitative increase. In addition, protein quality should be interpreted together with matrix‐dependent factors, including antinutritional compounds, protein–starch–fiber interactions, lipoprotein complexes, thermal processing effects, and changes in dough or batter functionality, all of which may influence protein bioaccessibility and end‐product quality.

The evidence reviewed in this work highlights that no single protein source can be considered universally optimal for all farinaceous matrices. Animal‐derived proteins generally provide high digestibility and biological value, but their application may be constrained by cost, sustainability, allergenicity, and matrix‐specific technological behavior. Plant‐based proteins offer relevant advantages in terms of sustainability, bioactive composition, and consumer demand; however, they often require amino acid complementation, antinutrient‐reducing pretreatments, and careful formulation to preserve technological performance. Insect and algae‐derived proteins represent promising alternatives because of their protein density, functional compounds, and environmental potential, yet their broader application still faces challenges related to sensory acceptance, regulatory harmonization, allergenicity, contaminant control, digestibility, and compatibility with gluten–starch networks.

## Nomenclature

AAS: amino acid score
ANFs: antinutritional factors
DIAAS: digestible indispensable amino acid score
EAAI: essential amino acid index
EAAs: essential amino acids
EFSA: European Food Safety Authority
GRAS: Generally Recognized As Safe
MPC: milk protein concentrate
PDCAAS: protein digestibility‐corrected amino acid score
PER: protein efficiency ratio
PPI: pea protein isolate
SFP: salmon fish powder
SPI: soy protein isolate
WAI: water absorption index
WBC: water‐binding capacity
WPC: whey protein concentrate
WPI: whey protein isolate
WSI: water solubility index

## Author Contributions

**P. Álvarez‐Arteaga**: conceptualization, investigation, writing – original draft, data curation. **M. A. Lazo‐Vélez**: conceptualization, funding acquisition, supervision, writing – review and editing. **L. Mateo Cordero‐Clavijo**: writing – original draft, writing – review and editing, supervision, conceptualization. **S. Paterson**: conceptualization, funding acquisition, writing – original draft, writing – review and editing.

## Funding

This research was supported by the NutriOmics Research Chair of the Universidad del Azuay (Ecuador), under project UDA 2025‐0137. This work was conducted under the Programa Iberoamericano de Ciencia y Tecnología para el Desarrollo (CYTED) through RED124RD0164. Samuel Paterson gratefully acknowledges the Comunidad Autónoma de Madrid for a predoctoral contract (PIPF‐2022/BIO‐24996).

## Conflicts of Interest

The authors declare no conflicts of interest.
